# Cerenkov luminescence imaging: physics principles and potential applications in biomedical sciences

**DOI:** 10.1186/s40658-017-0181-8

**Published:** 2017-03-11

**Authors:** Esther Ciarrocchi, Nicola Belcari

**Affiliations:** 1 Department of Physics “E. Fermi”, University of Pisa, Pisa, Italy; 2 INFN, section of Pisa, Pisa, Italy

**Keywords:** Cerenkov luminescence imaging, Nuclear medicine, Optical imaging

## Abstract

Cerenkov luminescence imaging (CLI) is a novel imaging modality to study charged particles with optical methods by detecting the Cerenkov luminescence produced in tissue. This paper first describes the physical processes that govern the production and transport in tissue of Cerenkov luminescence. The detectors used for CLI and their most relevant specifications to optimize the acquisition of the Cerenkov signal are then presented, and CLI is compared with the other optical imaging modalities sharing the same data acquisition and processing methods. Finally, the scientific work related to CLI and the applications for which CLI has been proposed are reviewed. The paper ends with some considerations about further perspectives for this novel imaging modality.

## Introduction

Cerenkov luminescence imaging (CLI) is an optical imaging modality to study charged particles of sufficient energy through the Cerenkov light they produce in biological tissue [1–3]. Cerenkov luminescence is emitted when a charged particle traverses a dielectric medium with a velocity greater than the phase velocity of light in the medium. The light is emitted on a cone around the particle direction, with a typical continuous spectrum proportional to the inverse wavelength squared. The range and the relatives intensities of the emitted wavelengths are limited by the material properties. In tissue, the emitted light is then highly scattered and absorbed before reaching the surface, and the tissue optical properties tend to favor the transmission of the red-infrared light, where the Cerenkov emission is minimal. Due to the low light level, the detection of Cerenkov luminescence typical requires the same type of imaging system of bioluminescence, that is a high sensitivity charge coupled device (CCD) coupled to a focusing optics and placed in a light-tight environment.

Even if CLI suffers of challenges such as the low light yield and the limited penetration depth, it has been proposed for several applications both in the preclinical and clinical fields. In fact, apart from these limitations, up to date CLI offers a valid tool to image *β* ^−^ radioisotopes, which can not be imaged with positron emission tomography (PET) and for which Bremsstrahlung imaging has not shown promising results yet [4, 5]. Bremsstrahlung imaging typically uses single-photon emission computed tomography (SPECT) equipment (i.e., a gamma camera) to detect the higher energy (compared to Cerenkov radiation) and broad spectrum radiation emitted by decelerating charged particles. Several studies have been performed using ^90^Y, as it emits almost purely *β* ^−^ particles which can not be imaged with PET. Although some of these studies have shown the potential of ^90^Y Bremsstrahlung imaging, for example for accurate dosimetry purposes [6], even in whole-body imaging [7], others have highlighted the limitations and challenges of this imaging modality [4, 5, 8]. In particular, the low count rate and the continuous spectrum of Bremsstrahlung radiation limit the sensitivity and the quantitative capabilities of Bremsstrahlung SPECT imaging. This, combined with the low spatial resolution and the high amount of scatter intrinsic in SPECT imaging, requires sophisticated correction methods for accurate dosimetry of activity levels [9, 10]. All these challenges suggest why Cerenkov radiation has been proposed as a possible alternative means to study isotopes such as ^90^Y.

CLI also offers a tool to image nuclear medicine probes with optical methods, which are much cheaper than conventional detectors for higher energy photons, and would therefore allow a larger spread of the imaging of these radioisotopes. Nonetheless, many radioisotopes are already approved for clinical use, thus allowing immediate translation to human applications.

Although CLI has been originally proposed for functional imaging of *β*-emitting radioisotopes [1–3, 11–13], other applications have later been suggested, such as Cerenkov-based surface dosimetry in external beam radiotherapy [14, 15] or the use in proton therapy for quality assurance and in vivo dosimetry [16]. In proton therapy, the high inherent spatial resolution of CLI compared to PET has been used to monitor the proton range by imaging the proton-induced positron distribution [17]. In external therapy, CLI has been utilized also for patient positioning and movement tracking [18]. Other applications suggested so far are Cerenkov luminescence endoscopy [19, 20] and Cerenkov luminescence guided tumor surgical resection [21]. The possibility of enhancing the Cerenkov signal by wavelength-shifting it to more penetrating longer wavelengths has also been evaluated [22, 23]. Another solution to increase the optical signal has been proposed in [24], by developing a hybrid light imaging technique that uses a liquid scintillator to assist CLI.

The investigation of this new imaging modality has also led to the exploitation of the Cerenkov light produced in media different than tissue in other biomedical contexts. One example is the utilization of the prompt Cerenkov emission in crystals used in positron emission tomography (PET), as a opposed to the delayed emission of scintillation light, with the goal of improving the time of flight performances of the imaging system [25]. Another example is the use of the Cerenkov light generated in plastic optical fibers for proton therapy dosimetry [26].

This review paper introduces the physical basis of the Cerenkov emission and then focuses on the light generation in tissue by radioisotopes. Some potential sources of spurious luminescence in tissue are also discussed. Then, the processes governing the transport of the emitted Cerenkov light in biological tissue are presented, followed by a brief description of the detector components of an optical imaging system and of their most relevant features for CLI applications. CLI is then compared with the other optical imaging modalities sharing the same data acquisition and elaboration techniques (bioluminescence and fluorescence imaging). Unless specified, all the educational figures shown in this paper were adapted from [27]. In the last part of this paper, the studies on CLI published up to date are reviewed, and some comments on CLI further perspectives are proposed, followed by a short conclusion.

## Review

### Cerenkov light production

Cerenkov radiation is prompt bluish-white light emitted when a charged particle passes in a dielectric medium with a velocity greater than the phase velocity of light in that medium. The charged particle induces polarization of the molecules in the medium, and radiation is emitted upon relaxation of these molecules. L’Annunziata [28] reports that Cerenkov radiation was first observed by M. Curie in 1910, but dedicated experiments were not performed until the 1930s, and in 1958 resulted in the award of the Nobel Prize to the three main investigators, P. Cerenkov, I. Frank, and I. Tamm. A detailed description can be found in [29], while more recent references are [30] or [31].

#### *Cerenkov relation*

**Fig. 1:**
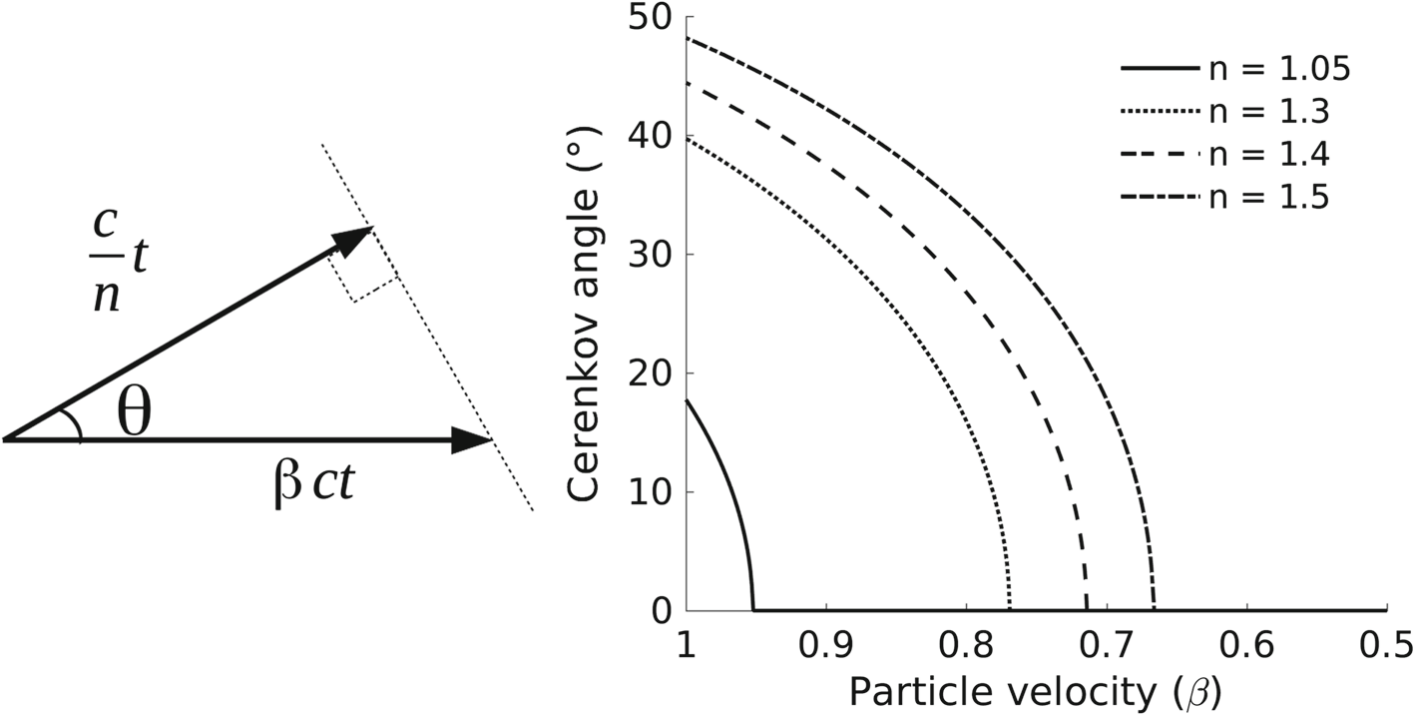
*Left*: Pictorial description of the Cerenkov emission. *Right*: Cerenkov angle as a function of the particle velocity, for different values of refractive index

The Cerenkov radiation is emitted on a cone around the particle direction. The characteristic emission angle *θ* depends on the velocity of the charge *β*=*v*/*c* (with *c* being the speed of light in vacuum) and the refractive index of the medium *n*, through the Cerenkov relation (Fig. 1, left):

1 $$ \cos{\theta} = \frac{1}{\beta n}  $$

Therefore the Cerenkov angle is reduced as the particle slows down in the medium, as shown in Fig. 1 (right) for different values of the refractive index, typical of air (*n*=1.05), water (*n*=1.33), tissue (*n*=1.4) and glass (*n*=1.5). The Cerenkov relation (1) has three consequences, due to the fact that *β* and cos*θ* are comprised between 0 and 1:

- maximum emission angle: $\theta _{max} (\beta \rightarrow 1) \rightarrow \arccos \left (\frac {1}{n}\right)$
- minimum emission velocity: $\beta _{min} (\theta = 0) = \frac {1}{n}$ (Cerenkov threshold)
- limit on refractive index: $n_{min} (\theta = 0) = \frac {1}{\beta }$

**Fig. 2:**
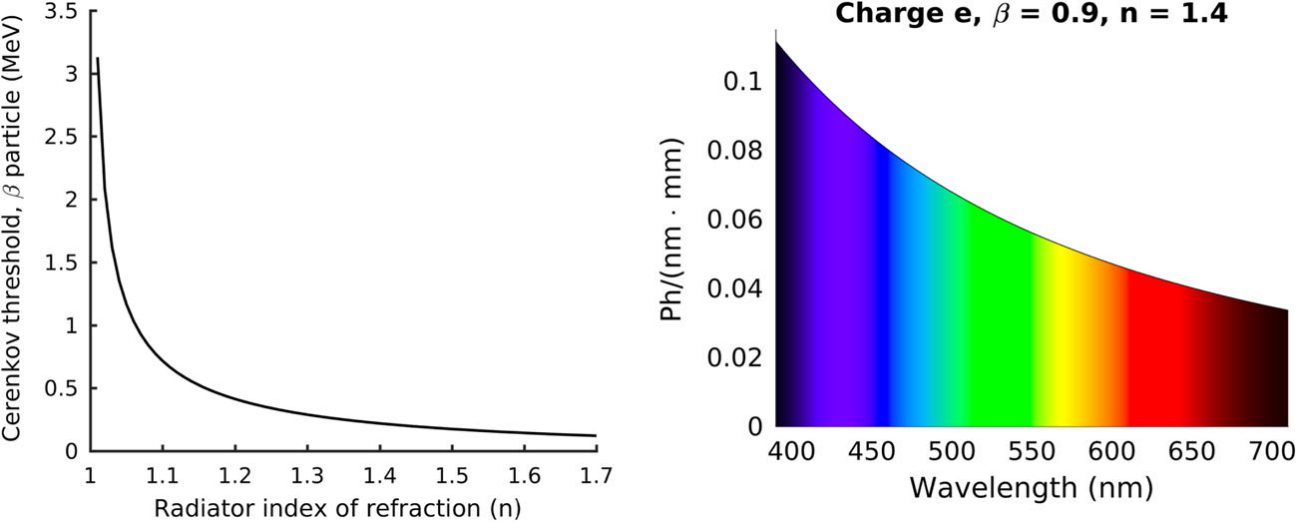
*Left*: Cerenkov threshold as a function of the refractive index of the radiator for a *β* particle (*m*_0_ *c* ^2^=0.511 MeV). *Right*: Number of Cerenkov photons emitted per unit path length and per unit wavelength by a particle with charge *e* and *β*=0.9 in a medium with *n*(*λ*)=1.4

The Cerenkov threshold can be expressed in terms of the kinetic energy of the charged particle with the following equation (using *E*=*T*+*m*_0_ *c* ^2^=*γ* *m*_0_ *c* ^2^):

2 $$ T_{min} = m_{0} c^{2} \left[\gamma\left(\beta_{min}\right) - 1\right]   $$

where *m*_0_ *c* ^2^ is the particle rest mass, $\gamma = \sqrt {\frac {1}{1 - \beta ^{2}}}$ and *E* is the total energy of the particle. For water (*n*≃1.33 in the visible range) and for average soft tissue (*n*≃1.4) the Cerenkov threshold for a *β* particle is 264 and 219 keV, respectively. The threshold kinetic energy for the Cerenkov emission by a *β* particle with rest mass *m*_0_ *c* ^2^=0.511 MeV is shown in Fig. 2 (left) as a function of the refractive index of the medium, showing that in media with a higher refractive index the Cerenkov threshold is lower, as suggested first in [32]. Due the dispersion of the refractive index as a function of wavelength (*dN*/*d* *λ*), different wavelengths will have a slightly different Cerenkov threshold. For water, the variation of the refractive index over the visible wavelength range (*λ*=400−700 nm) is less than 7%, giving a variation of the Cerenkov threshold of less than 3%. The dispersion, together with the energy loss and the multiple scattering of the charged particle in the medium, causes a spread of the spatial distribution of the emitted light around the Cerenkov angle and a finite duration of the light flash. However, these effects are not relevant for CLI applications because the emission of the primary charged particles is itself continuous in time and isotropic in space.

#### *Cerenkov emission spectrum*

The Cerenkov emission spectrum is peaked in the ultraviolet-blue and follows the one over wavelength squared dependence by the Frank and Tamm formula. The number of photons emitted per unit path length by a particle with charge *e* [30] is:

3 $$ \frac{dN}{dl} = 2\pi\alpha \int_{\beta n > 1} \left(1 - \frac{1}{\beta^{2} n^{2}(\lambda)}\right) \frac{1}{\lambda^{2}} d\lambda   $$

where *α*≃1/137 is the fine structure constant. In terms of number of photons emitted per unit path length and per unit wavelength, (3) becomes

4 $$ \frac{dN}{dl \cdot d\lambda} = 2\pi\alpha \left(1 - \frac{1}{\beta^{2} n^{2}(\lambda)}\right) \frac{1}{\lambda^{2}}  $$

An example is shown in Fig. 2 (right) for an electron with *β*=0.9 and *n*=1.4, constant for all wavelengths. Due to the non-linear relationship between energy and wavelength (*E*=2*π* *hc*/*λ*), the energy spectrum of the emitted radiation appears flat:

5 $$ \frac{dN}{dl \cdot dE} = \frac{\alpha}{\hbar c} \left(1 - \frac{1}{\beta^{2} n^{2}(E)}\right)  $$

The formula for the Cerenkov spectrum gives an infinite light production. In reality, it has two cut-offs, at long wavelengths due to the medium self-absorption and at short wavelengths in the X-ray region, where *n* becomes less than unity (in the so-called *region of anomalous dispersion*) and the Cerenkov relation (1) is no longer satisfied [29].

As an example, Table 1 summarizes some figures of merit of the Cerenkov emission in water and tissue. The Table reports the average refractive index of the two materials in the (400–1000) nm range, the maximum angle of Cerenkov emission, the energy threshold for Cerenkov production and the number of photons per mm produced in the (400–1000) nm range. Due to the higher refractive index, the Cerenkov threshold in tissue is lower than in water, thus the number of photons produced per unit path length is higher. For water, more examples can be found in [33], which reports some calculated total number of Cerenkov photons produced in the range 250–600 nm in water by electrons of different energies.

**Table 1:** Some figures of merit of the Cerenkov emission in water and soft tissue

	Water	Tissue
*n*, average in (400–1000) nm	1.33	1.4
*θ*_*max*_(*β*→1)	41°	44°
*T*_*min*_(*θ*=0), *β* particle	264 keV	219 keV
$\frac {dN}{dl} (400-1000\:\text {nm}), \: \beta \rightarrow 1$	20 ph/mm	23 ph/mm

#### *Comparison with other forms of energy loss*

**Energy loss for ionization** To have an idea of the weakness of the Cerenkov radiation, it is useful to note that for a minimum ionizing particle the rate of energy loss per unit path length (*dE*/*dx*) due to Cerenkov emission is about 0.1% of that due to ionization [34]. In terms of number of photons per unit path length (*dN*/*dx*), in [30], it is estimated that the light output of Cerenkov emission is approximately two orders of magnitude weaker than that of a plastic scintillator.

**Bremsstrahlung radiation** The Cerenkov effect should not be confused with Bremsstrahlung radiative emission. The Cerenkov emission depends on macroscopic properties of the medium through the index of refraction, while Bremsstrahlung emission is the result of an interaction of the moving charged particle with the Coulomb field of the nucleus of the medium. If the charge travels close to the nucleus, it is slowed down and emits radiation. The energy of this radiation is comparable to that of the moving charge, and therefore much higher than that of the Cerenkov radiation. Jelley [29] estimates that, for an electron traversing 1 cm of water with a kinetic energy of 100 MeV, the ratio between the Bremsstrahlung signal and the Cerenkov signal in the visible range *λ*=400−500 nm is of the order of 10 ^−5^.

#### *Cerenkov luminescence production in biological tissue*

A significant fraction of the *β* particles emitted by radionuclides commonly used in biomedical imaging have an energy above the Cerenkov threshold in tissue (Table 1), and therefore are able to produce a detectable amount of Cerenkov radiation. Table 2 summarizes some useful information for a few common radionuclides: the type of decay, the half life, the endpoint energy and the fraction of *β*-particles above the Cerenkov threshold in water (assuming *n*=1.33) and tissue (*n*=1.4). The endpoint energy determines the fraction of *β* particles that are able to produce Cerenkov radiation. ^18^F-FDG (Fluorine-18 fludeoxyglucose or fluorodeoxyglucose) is used as a non-specific tracer to measure glucose metabolism (mainly for oncological studies), Iodine-131 (^131^I) is a radiotherapeutic used in nuclear medicine to treat thyroids affected by various diseases, while Yttrium-90 (^90^Y) is widely used in the radiation therapy of cancer (mainly in the liver), but also to treat joints affected by rheumatoid arthritis. The study described in [35] surveyed several radionuclides to characterize their Cerenkov light emission, showing in particular that ^131^I produces the smallest signal among the radioisotopes listed so far, followed by ^18^F and ^90^Y, as indicated in Table 2.

**Table 2:** Characteristics of some radioisotopes

Isotope (decay)	*t*_1/2_	Endpoint energy (keV)	Fraction above CT (%)	
			water	tissue
			(*n*=1.33)	(*n*=1.4)
^18^F (*β* ^+^)	109.8 min	633	42	58
^90^Y (*β* ^−^)	64.1 h	2280	90	93
^131^I (*β* ^−^)	8.02 days	606	25	37

CT = Cerenkov threshold

**Fig. 3:**
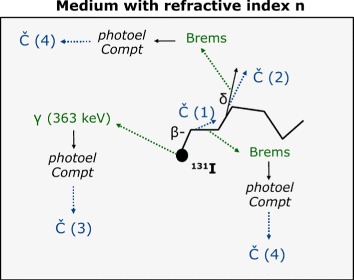
Pictorial description of the different processes that can lead to Cerenkov emission. Abbreviations: Brems = Bremsstrahlung, photoel = photoelectron, *δ* = *δ* ray, C̆ = Cerenkov radiation. Adapted from [69]

Cerenkov luminescence production in biological tissue is a complicated process, resulting from several types of interaction. Let us take for example the simplified decay scheme of ^131^I, and in particular let us consider the most probable decay. The ^131^I decay produces an electron (with maximum energy of 606 keV) and one 363 keV *γ* photon. As summarized in Fig. 3, Cerenkov radiation can be produced directly by the primary *β* particles (1) or by secondary electrons with kinetic energy above the Cerenkov threshold. These secondary electrons can be *δ* rays produced by the primary *β* particles (2), photoelectrons or Compton electrons produced by the *γ* ray (3) or by Bremsstrahlung radiation (4). Similar considerations apply also to ^18^F, for which the contributions due to the two annihilation photons should also be accounted for, while the process is much simpler for ^90^Y, as it almost a pure *β* ^−^ emitter. In applications like proton-therapy, the scheme can be even more complex, as described in [16]. However, when using biomedical radioisotopes, most of the high energy photons interactions are Compton events, thus the energy transferred to the electron is most often below the Cerenkov threshold. To confirm this, Monte Carlo simulations performed by [36] have shown that the main contribution comes from the primary *β*-particles, that emit the Cerenkov radiation very close to the point where the radioisotope decays.

**Fig. 4:**
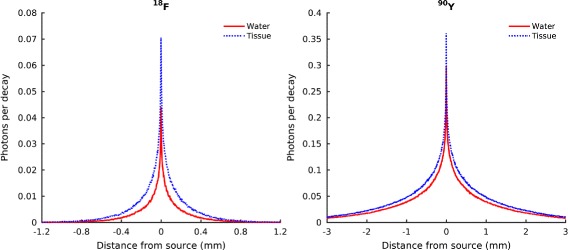
Cerenkov emission range, defining the CLI intrinsic limit to the spatial correlation between the source position and the position of production of the Cerenkov radiation, much like the positron range for positron emission tomography. Simulated results were obtained including all the contributions to Cerenkov light production (1–4) of Fig. 3

Figure 4 shows the distribution of the Cerenkov light production around a point source, simulated with Geant4, for ^18^F (left) and ^90^Y (right), in water (*n*=1.33, solid line) and in tissue (*n*=1.4, dotted line). The distributions were obtained considering in the simulations all contributions (1–4) in Fig. 3. For both radioisotopes, the spread of the light production increases from water to tissue due to the reduction in the Cerenkov threshold as a consequence of the increased refractive index (from approximately 264 keV to 219 keV, see Table 1). The different *x* and *y* scales for the two radioisotopes emphasize two opposing aspects. Due to its lower energy, the ^18^F production is more limited in space but also smaller in intensity. On the other side, ^90^Y has a worse spatial resolution since its light distribution is broader (the electrons emitted by ^90^Y travel more than the positrons of ^18^F before losing energy to the point of being below the Cerenkov threshold), but it can be detected better due to the stronger signal.

**Fig. 5:**
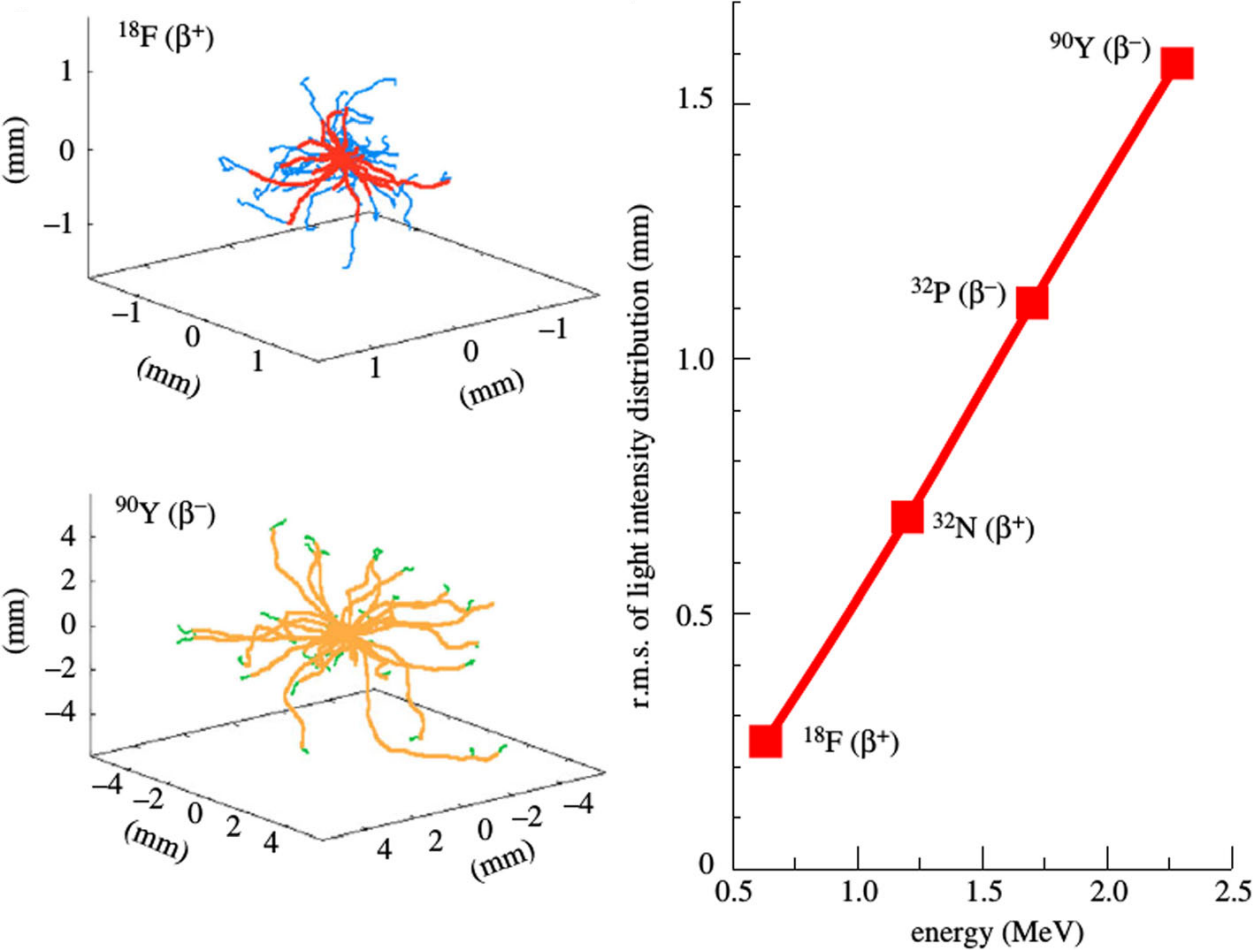
*Left*: Cerenkov production range simulated by [32], considering only the contribution of the primary *β* particles (1) in Fig. 3. *Right*: simulated spread in the Cerenkov light production as a function of the *β*-particle endpoint energy, for a few radionuclides (both with permission by [32])

For all radioisotopes, the Cerenkov emission by the primary *β* particles only is confined within a few millimeters around the radionuclide position. This can be seen clearly in the simulated results of Fig. 5 by [32]. The left part of the figure shows some *β*-particle tracks emanated from a point source of ^18^F (top) and ^90^Y (bottom) in water, and the change in color indicates the point where the energy of the particles goes below the Cerenkov threshold. The right part of the figure shows the root mean square spread of the Cerenkov light production for a few radioisotopes, plotted as a function of the endpoint energy of the *β*-particle.

The additional contribution from secondary particles (see Fig. 3) degrades the correlation between the source position and the Cerenkov emission, producing light farther away from the source, but this contribution is relevant only for high energy PET radioisotopes such as ^89^Zr [36]. It should be noted that the intrinsic limit to the spatial correlation of the Cerenkov light production with the source position is smaller for CLI than for PET, where it is determined by the positron range. However, in a real measurement this advantage is negligible because the width of the spatial distribution of the detectable light is largely dominated by scattering in tissue.

From what said so far about the phenomenon of Cerenkov production in tissue by *β* particles, it is evident that the detectable signal in a CLI experiment depends on several factors. Not only the refractive index determines the intensity of the light production, but also the radioisotope being imaged (determining the fraction of *β* particles above the Cerenkov threshold), and the density and geometry of the medium where the radioisotope is located (determining the distance over which the *β* particles reach the Cerenkov threshold due to their energy loss and the fraction of *β* particles that escape the volume before having lost enough energy to be below the Cerenkov threshold). We now briefly present some sources of luminescence that might contribute to the reduction of the signal-to-noise ratio by increasing the background luminescent noise, and we will discuss next how the transport in tissue also affects the final detectable Cerenkov signal.

#### Potential sources of spurious luminescence in tissue

**Bremsstrahlung** As previously discussed, the contribution of Bremsstrahlung emission in the visible wavelength range is quite small. In addition, the probability of significant Bremsstrahlung radiation production in tissue is low due to the low atomic number [1].

**Autofluorescence and radioluminescence** The tissue itself also plays the role of a contrast agent by naturally emitting luminescence upon external excitation. Autofluorescence is the absorption and re-emission of light of longer wavelength due to small amounts of fluorophores inherently contained in biological tissues [37]. This type of luminescence can be either an unwanted background [38] or be intentionally exploited for imaging purposes to distinguish normal from cancerous cells [39]. The relevance of the autofluorescence induced by Cerenkov emission has not been investigated yet, but [38] assumes that its contribution should be negligible for CLI. Radioluminescence is light production in a material by bombardment with ionizing radiation and the role of radioluminescence in tissue as a source of noise for CLI has not been investigated yet. ^1^

**Black body radiation** Another potential problem when performing optical imaging could be the overlap with the black body emission in the infrared (IR), also called thermal radiation. However, Wien’s displacement law predicts the maximum emission at *λ*=2.9/*T*·10^6^ nm · K, which for a body temperature of *T*≃310 K corresponds to approximately 9350 nm, far away from the Cerenkov emission range. Yet it should be noted that black body radiation is successfully used for thermal imaging using infrared cameras.

### Cerenkov light transport in tissue

After being produced in biological tissue, Cerenkov radiation undergoes several interactions before escaping the tissue and eventually reaching the detector. Proper modeling of these interactions is essential for an accurate quantitative determination of the activity level responsible of the Cerenkov emission. The optical properties of the tissue components that determine the propagation of light are the scattering and absorption coefficients and the tissue inhomogeneity in the refractive index, which causes reflection and refraction of the light. Most biological tissues are characterized by strong optical scattering and weak absorption [40] and are therefore considered diffusive. These interactions are the biggest challenge in any optical imaging modality, because they severely limit both the spatial resolution (mainly due to scattering and discontinuity of the refractive index) and the sensitivity (due to absorption). Refraction at the interface between tissue and air may also impact the total number of detected photons. This is particularly crucial for CLI, where the signal is already weak at production and the emission is on a broad spectrum, over which the optical properties vary much.

Different studies have been performed to determine the optical properties of the various biological tissues (see for example [41] or [42]), but available data over the whole visible spectrum are rather limited due to the strong dependence on the wavelength, the tissue preparation and the measurement technique. Jacques [43] has provided empirical formulas to calculate the tissue optical properties for a given wavelength from factors such as the content of water, fat and hemoglobin. However, the high variations in the experimental data of different groups and studies seriously complicate the modeling of the optical transport.

**Fig. 6:**
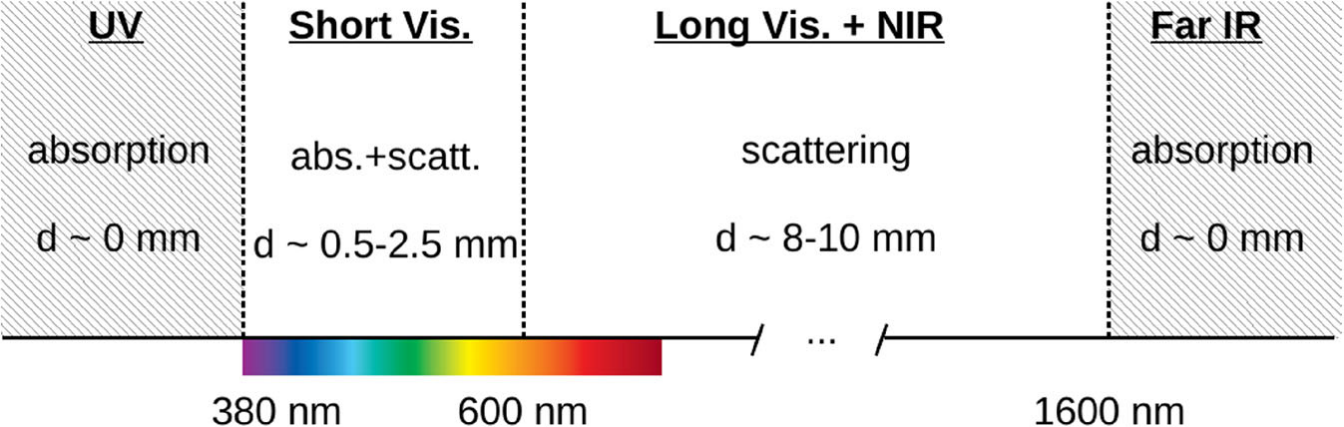
Summary of the behavior in tissue of optical radiation of different wavelength, from data in [42]. The penetration depth is indicated as *d*

Based on [42], the light behavior in tissue can be summarized with the scheme shown in Fig. 6. Light is strongly absorbed in the ultraviolet (*λ*<380 nm) and far infrared (*λ*>2000 nm) spectral regions. Short wavelength visible light penetrates as deep as 0.5–2.5 mm, with an *e*-fold decrease of intensity with penetration in tissue, due to the combined effect of absorption and scattering. In the 600–1600 nm wavelength range, scattering dominates over absorption, and light can penetrate up to 8–10 mm.

#### *Absorption*

The absorption coefficient *μ*_*a*_ defines the probability per unit path length of photon absorption in a medium. Its reciprocal is called mean absorption length. In a pure absorber (no scattering) of thickness *x* with absorption coefficient *μ*_*a*_, the attenuation of a collimated beam of light with incident intensity *I*_0_ is described by the Lambert-Beer law:

$$I(x) = I_{0} \mathrm{e}^{-\mu_{a}x} $$

The absorption coefficient *μ*_*a*_ of a tissue is the sum of the contributions from all absorbing chromophores within the tissue, weighted with the concentration of each absorber *c*_*i*_:

$$\mu_{a} = \sum_{i}{c_{i} \mu_{a,i}} $$

**Fig. 7:**
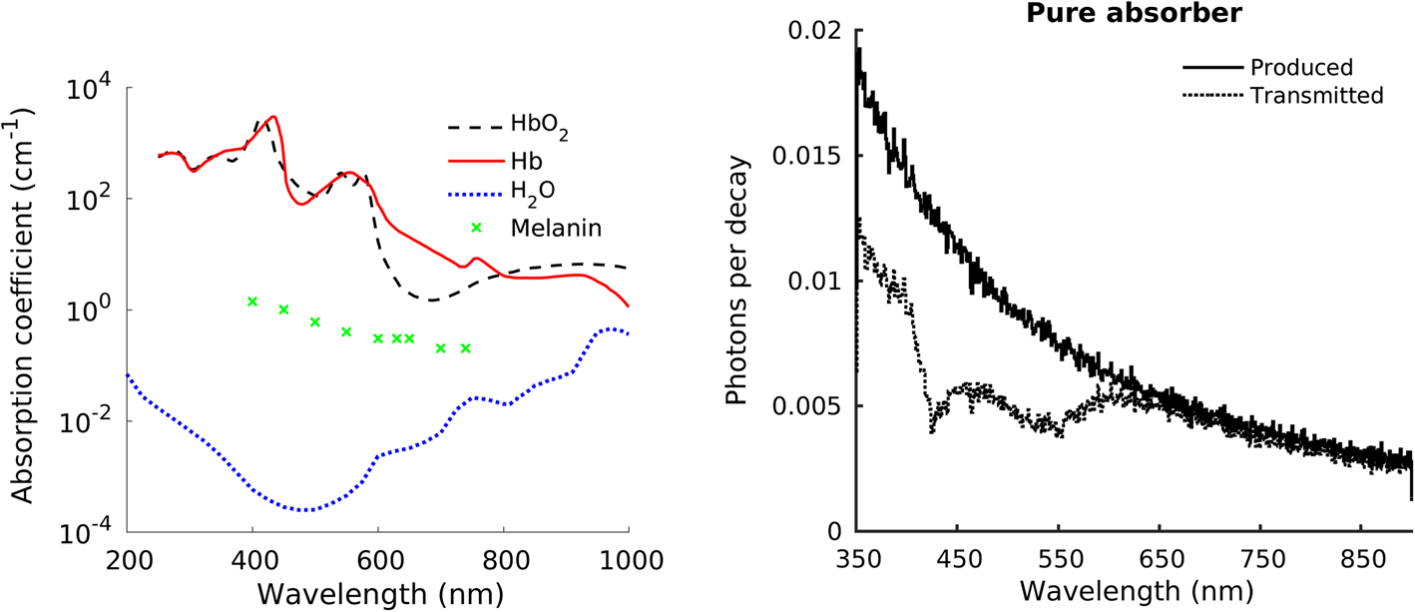
*Left*: Absorption spectra of tissue components. Data for both oxygenated and deoxygenated hemoglobin were taken from [44], water data by [45] and melanin data by [46]. *Right*: Monte Carlo simulation of the modification of the Cerenkov spectrum in 1 cm of a typical tissue-like pure absorber (*dotted line*), with the original Cerenkov emission spectrum as a reference

In biological tissue, optical absorption is due mainly to hemoglobin, melanin and water. The absorption spectra of these tissue components are shown in Fig. 7 (left), and they were obtained form different sources: the absorption coefficient of hemoglobin (in both its oxygenated and deoxygenated forms) was tabulated by Prahl [44], using data by Gratzer and Kollias; the absorption of water was measured by Hale and Querry in 1973 [45]; the data for melanin were tabulated by Lister et al., [46] using data by Chan. et al., [47]. As shown in Fig. 7 (left), absorption is stronger for ultraviolet light than for longer wavelengths. Minor contributions from other tissue components usually do not perturb significantly the optical transport and therefore are not significant if one is interested only in understanding light penetration. Absorbed light is converted into heat or luminescence, or it is consumed in photochemical reactions [40].

In CLI, absorption causes the loss of the peculiar spectral distribution of the Cerenkov light (proportional to the inverse wavelength squared). To help visualize this effect, a Monte Carlo simulation was performed in Geant4. Figure 7 (right) shows an example of the effect on the Cerenkov spectrum of pure absorption after traversing 1 cm of biological tissue (the optical properties of chicken breast measured by [48] were used for this example), indicating a stronger absorption at short wavelengths, and an almost unchanged shape of the spectrum at long wavelengths.

#### *Scattering*

Optical scattering originates from light interaction with biological structures of different size (from a few tenths of nm to hundreds of *μ*m) and shape. Photons are scattered more strongly by structures whose size matches their wavelength and whose refractive index mismatches that of the surrounding media [40]. Scattering can be elastic or inelastic (Raman). In an elastic scattering event, the trajectory of the photon is deviated. In an inelastic scattering event, also the wavelength of the photon is changed according to the conservation of energy. Raman inelastic scattering is known to be weak compared to the elastic component (the study in [49] reports as a typical value that only one in 10^8^ photons undergoes Raman scattering in tissue) and is therefore usually neglected when treating tissue optics.

**Fig. 8:**
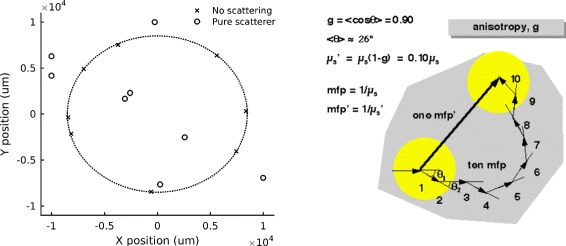
*Left*: Monte Carlo simulation of the effect of pure scattering on the transmitted light distribution: positions of the Cerenkov photons belonging to the same *vertex* after traversing 1 cm of either a non-scattering material (*crosses*, producing the Cerenkov cone indicated by the *dashed circle*) or a tissue-like scattering material (*circles*). *Right*: Difference between the scattering and the reduced scattering coefficients (with permission from www.omlc.org, accessed on 21/12/2016)

Scattering of Cerenkov light causes the loss of the peculiar angular distribution of the photons (on a cone around the particle direction). Figure 8 (left) reports simulation results to show the effect of pure scattering, using the optical properties of chicken breast measured by [48]. The crosses (with the dotted line identifying the cone) are the positions of the Cerenkov photons belonging to the same *vertex* after traversing 1 cm of non-scattering material. The circles are instead the positions of these photons after interacting in a pure scatterer: the peculiar angular distribution of the Cerenkov light is no longer visible.

There are three parameters that describe the scattering phenomenon and that can be measured experimentally.

**Scattering coefficient** The scattering coefficient *μ*_*s*_ defines the probability per unit path length of photon scattering in a medium. Its reciprocal is called scattering mean free path.

**Anisotropy parameter** The anisotropy parameter *g* is defined as the average cosine of the scattering angle *θ*:

6 $$ g = \langle \cos{\theta}\rangle = \int_{0}^{\pi} p(\theta) \cdot \cos\theta \cdot 2\pi \sin \theta d\theta   $$

The parameter *g* varies between -1 and 1: *g*=0 corresponds to isotropic scattering (Rayleigh), *g*=1 to total forward scattering and *g*=−1 to total backward scattering. For biological structures, the average anisotropy parameter is *g*≃0.9, denoting a high probability of the photon to be scattered in the forward direction in each scattering event. However, after multiple scattering events the propagation direction is gradually randomized [50], which is the reason why the Rayleigh approximation holds (see below).

**Reduced scattering coefficient** For diffusive media like biological tissue (*μ*_*s*_≫*μ*_*a*_), the anisotropy parameter allows to define the reduced scattering coefficient *μ* *s*′ as:

7 $$ \mu_{s}' = \mu_{s}(1 - g)   $$

The difference between the scattering coefficient *μ*_*s*_ and the reduced scattering coefficient *μ* *s*′ is illustrated in Fig. 8 (right). With the reduced scattering coefficient, a random walk made by many small anisotropic steps can be described with less equivalent big steps involving isotropic scattering. For example, for tissue with *g*=0.9, we have *μ* *s*′=0.1*μ*_*s*_, therefore the reduced mean free path *MFP* ^′^=1/*μ* *s*′ is ten times longer than the mean free path *MFP*=1/*μ*_*s*_. This is of course valid only for diffusive media, where there are many scattering events before an absorption event.

The reduced scattering coefficient is not only convenient to reduce the computational cost when modeling tissue optics, but it is also easier than the scattering coefficient to be determined experimentally. In fact, the scattering coefficient *μ*_*s*_ is usually measured by a collimated transmission measurement *T*_*c*_ through a slab of tissue of thickness *l*, so that *μ*_*s*_=− log*T*_*c*_/*l*, and requires a very thin slab (of the order of the scattering mean free path which is less than 100 *μ*m) to avoid multiple scattering events [43]. The measurement of the reduced scattering coefficient *μ* *s*′ is instead performed by diffuse light measurements using thicker tissue slabs, which are easier to obtain. The two coefficients allow to determine experimentally the coefficient as *g*=1−*μ* *s*′/*μ*_*s*_, otherwise difficult to measure directly.

The scattering properties vary strongly among different biological tissues, and they are heavily dependent on the light wavelength and on the measurement conditions. Therefore, a summary of the values of these parameters is not presented in this paper. However, a detailed review and additional references can be found for example in [43].

Scattering of light by a spherical particle can be modeled exactly by the Mie theory, which reduces to the Rayleigh theory for light interacting with particles whose size is much smaller than the incoming wavelength. The following paragraph briefly describes the Rayleigh approximation, which might be useful when modeling the forward problem of the transport of Cerenkov light in tissue. The reader is invited to refer to the published literature, such as [40, 42] for more details and for a description of the more general Mie theory.

**Rayleigh scattering** The Rayleigh theory models the elastic scattering of a plane monochromatic wave by a single particle whose size is much smaller than the wavelength $n_{rel} \left (\frac {2\pi a}{\lambda }\right) \ll 1 $, where $n_{rel} = \frac {n_{s}}{n_{b}}$ is the relative refractive index of the scattering particle (*n*_*s*_) with respect to the background (*n*_*b*_) and $\left (\frac {2\pi a}{\lambda }\right)$ is the *size-parameter*, that depends on the size of the scatterer *a* and on the wavelength *λ* of the incident light. For visible and NIR light and for *n*_*rel*_≃1.05−1.1 as in tissue, the Rayleigh limit corresponds to *a*<(12−14) nm, and is strictly valid only for subcellular structures [42].

In a Rayleigh scattering interaction, only the direction of the incident wave is changed, while its wavelength is left unchanged. For unpolarized incident light *I*_0_ of wavelength *λ*, the distribution of the scattered light intensity is given by:

$$I(t,\theta) = \frac{\left(1 + \cos^{2}{\theta}\right) |\alpha|^{2}}{2r^{2}} \left(\frac{2\pi n_{b}}{\lambda}\right)^{4} I_{0} $$

where *θ* is the scattering angle, *r* is the distance from the scatterer, *α* is the polarizability of the particle (see below) and *n*_*b*_ is the refractive index of the background medium. This strong wavelength dependence is responsible of the stronger scattering of blue light with respect to red light. The polarizability of a sphere with radius *a* is given by $\alpha = \frac {n^{2}_{rel} - 1}{n^{2}_{rel} + 2}a^{3}$.

#### *Reflection and refraction*

When an optical photon finds a discontinuity of the refractive index (an optical interface), it has a certain probability to be reflected back or to be transmitted (refracted). These probabilities are calculated with the Fresnel equations and depend on the refractive indexes of the materials forming the interface and on the polarization of the incident photon [51].

The refractive index of tissue ranges between 1.35 and 1.7 (Table 3). For soft tissue, a refractive index *n*=1.4 is usually considered [52], but variations have been reported from sample to sample, place to place and time to time [43].

**Table 3:** Average values of refractive index of some tissue components

Component	Refractive index	Reference
Melanin	1.6–1.7	[40]
Adipose tissue	1.45	[52]
Kidney	1.417	[52]
Liver	1.367	[52]
Average soft tissue	1.4	[40]

### Detectors for CLI

Cerenkov luminescence imaging is typically performed in a light-tight environment with a system comprising a sensitive charge-coupled device (CCD) and a high aperture lens to increase the light collection efficiency. The most used imaging systems belong to the IVIS series [1–3, 11, 12, 22, 24, 32, 36, 53–58], but also custom-built systems have been employed [13–15, 17, 18, 21, 23, 59–62]. While IVIS imaging systems have been utilized mainly for in vitro and small animal imaging, custom systems have been built, featuring either intensified CCD cameras (ICCD) or electron-multiplying ones (EMCCD), for microscopic imaging, for phantom studies to test the feasibility of CLI applications and also for a few clinical human studies. Apart from the IVIS series, there are other commercially available optical imaging systems which could be adapted for CLI measurements. A list of some fluorescence imaging systems can be found in [63].

#### *Choice of the photodetector*

To optimize the detection of Cerenkov light, long exposure times and a completely dark environment are required. A photodetector for CLI should have high quantum efficiency over the wavelength range transmitted by the tissue being imaged, high sensitivity and low noise. Back-thinned back-illuminated CCD sensors are typically employed to increase quantum efficiency, while to reduce dark noise the CCD sensor is usually cooled down to temperatures as low as −90° C or even less.

In electron multiplying CCDs (EMCCDs, [64]), the electrical signal produced by the light impinging on the CCD sensor is amplified prior to readout in a so-called multiplication register [65]. The use of EMCCDs is suggested for low light levels and when short exposure times are required (dynamic imaging). If the signal intensity is high enough (i.e., more than 10 photons per pixel) or if long exposure times can be used, a conventional CCD provides the highest SNR.

Intensified CCD cameras (ICCD) instead increase sensitivity by means of an image intensifier screen placed before the CCD sensor. The incoming light hists the photocathode at the entrance surface of the screen, producing photoelectrons. These photoelectrons are then accelerated towards a micro-channel plate, where they are multiplied and accelerated again towards a phosphor screen which converts them back to an optical signal that is focused onto the CCD sensor [66]. Like EMCCDs, ICCDs are more sensitive than conventional CCD sensors for very low light levels.

Another aspect worth noting is the spectral composition of the Cerenkov signal. Although the emission is peaked in the UV-blue range, this part of the spectrum is more strongly attenuated as the source depth in tissue increases. Therefore, as suggested also in [67], photodetectors should be optimized for UV-blue light for surface imaging, but should be more sensitive to longer wavelengths for deeper sources. In this respect, in [68] it was estimated that the number of detected Cerenkov photons could be increased by up to 35% by choosing a CCD camera with quantum efficiency peaked in the NIR range.

#### *Choice of the optics*

Choosing the right lens for a specific application is usually a complicated task, because there are several factors that play a role in determining the imaging settings (e.g., the focal length, the aperture and the minimum working distance). Nonetheless, these factors are cross-correlated and constrain each other (for example because a longer focal length typically determines a longer minimum focusing distance, which in turn limits the allowable field of view).

In CLI the main goal is to maximize the amount of collected light, therefore low f-number lenses (corresponding to high aperture values) tend to be preferred. The f-number, usually denoted as *f*/*#*, is the ratio of the lens focal length to the diameter of its entrance pupil, which in a lens mount can be adjusted with an integrated iris. Increasing the *f*/*#* by a factor of $\sqrt {2}$ halves the area of the aperture, decreasing the light throughput of the lens by a factor of 2.

However, the f-number alone is not enough for the lens selection, because also a large diameter or a shorter distance (typically corresponding to a shorter focal length) increase the light collection efficiency. Despite this, usually the specific imaging application determines the field of view (FOV) to be imaged, therefore this is the fixed parameter that constrains the others. For a given size of the FOV, the light collection efficiency depends only on the lens f-number and on the sensor area. In particular, this efficiency is inversely proportional to the square of the f-number and depends linearly on the sensor area. Other limiting factors which should be considered are the minimum focusing distance, which poses a limit to the maximum allowable FOV, and the fact that small f-numbers imply small depths of field. A typical lens for optical imaging has a focal length of 25 mm and f-number f/0.95.

Finally, in the same way as quantum efficiency is crucial for the photodetector selection, also the wavelength-dependent transmission and the level of impurities (resulting in background luminescent emission) of the optical components should be carefully chosen. As an example, in [67] the use of fused silica rather than Schott-75 glass was suggested to improve sensitivity. In addition, long-pass filters have been employed for deeply located sources to suppress the contribution of short wavelength Cerenkov photons produced superficially (farther away from the source).

#### *Other detection systems for CLI*

CCDs are *integrating* devices, meaning that the signal produced is a measure of the total detected signal, integrated over a certain amount of time. One group has used a photomultiplier tube coupled to fiber optics for proton-therapy dosimetry [26], while in [69] digital silicon photomultipliers (SiPM) have been proposed as an alternative technology to CCDs. SiPMs are *single photon sensitive* devices, in the sense that they produce an output signal that allows to discriminate each detected photon. This behavior, opposite to the integrating one of CCDs, might be exploited for a more quantitative measurement of the number of detected photons, which in turn could be quantitatively correlated to the source activity.

### CLI and other optical imaging modalities

Cerenkov luminescence imaging relies on optical imaging methods to study the same radionuclides as used in PET and SPECT, inheriting from conventional optical imaging (bioluminescence and fluorescence imaging) the detection system, the image processing and reconstruction algorithms, as well as the challenges and limitations due to limited penetration depth of visible light. For these reasons, it seems natural to compare CLI with these two imaging modalities, highlighting the common features as well as the differences between the techniques.

**Fluorescence imaging** Fluorescence imaging uses fluorescent chromophores that are injected into the subject under study and activated with an external excitation [70]. Since the lifetime of the excited state of the fluorescent molecules is short (of the order of the nanosecond or less), the activation can be repeated enabling high count rates and sensitivity. The light emitted by the contrast agent is shifted towards more penetrating longer wavelengths and is imaged with an optical system. The need for an external excitation light causes a noise floor of autofluorescence and backscattered excitation light that is only partially rejected by filtering out unwanted wavelengths. Fluorescence imaging is currently used in surgery to identify the tumor volume as an aid in its resection [71]. Up to date, the only clinically approved fluorescent contrast agents are fluorescein, methylene blue and indocianine green (ICG).

**Bioluminescence imaging** Bioluminescence is the process of photon emission upon oxidation of luciferin by luciferase [39]. Therefore luciferin, if injected into the subjects, acts as a biomarker of gene expression, in particular by tumor cells, allowing in vivo tumor imaging. However, its applications are limited only to cells expressing luciferase. Bioluminescence imaging employs a spontaneous and single emission process on a limited wavelength range (500–565 nm, [70]). Even if there is no spurious emission due to an external excitation source as in fluorescence imaging, background autoluminescence from the bulk of the animal may limit the ability to detect bioluminescent probes [72].

#### *Comparison with CLI*

**Fig. 9:**
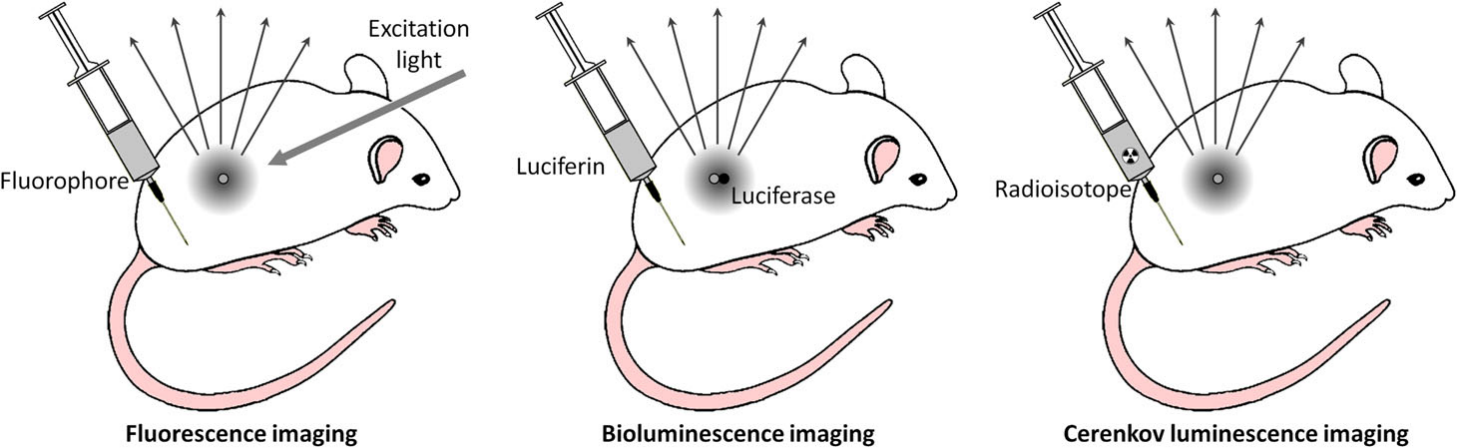
Overview of optical imaging modalities: fluorescence imaging (*left*), bioluminescence imaging (*center*) and Cerenkov luminescence imaging (*right*). This figure was conceived taking inspiration from both [70, 132]

The differences between these optical imaging modalities and CLI are summarized in Fig. 9. Fluorescence imaging requires an external excitation light and often a fluorescence agent is injected to enhance contrast (left). Bioluminescence relies on the injection of luciferin and on the presence of cells expressing luciferase (center). The CLI signal is produced spontaneously by a *β*-emitter in a medium with refractive index greater than air, such as tissue (right).

To try comparing quantitatively the detectable signal with these three techniques, the typical small animal photon count rates reported in [70] are of the order of (1-3) ·10^4^ cps for CLI, 10^3^−10^5^ cps for bioluminescence imaging, and greater than 10^8^ cps for fluorescence imaging. This relative comparison is in agreement with the description by [38] for CLI and the three clinically approved fluorescent probes, which reports that the typical emitted photon fluxes in CLI are two orders of magnitude smaller than those produced by ICG and methylene blue, and more than three orders of magnitude weaker than the intensity of the emission of fluorescein. The effect of tissue attenuation (higher in the Cerenkov emission range than in the range of emission of the fluorescent probes) causes an additional difference of one order of magnitude in the detectable signals when adding 1 mm of scattering tissue. However, one aspect that is worth noting is that bioluminescence and fluorescence imaging uses have so far been limited mostly to the preclinical field. This is due not only to the limitations in depth-penetration of optical radiation, but also to toxicity issues and long washout periods of optical probes, which restrict the utilization in human studies. On the other side, there are many clinically approved radioactive tracers and pharmaceuticals, which could be readily imaged in the clinic with CLI [73].

#### *Optical tomography*

As for tomographic imaging, the goal is to reconstruct the interior distribution of photons from measurements at the tissue surface, thus requiring models of the light transport for quantitative image reconstruction [70]. The solution of the inverse problem usually relies on the diffusion equation, an approximation of the radiative transfer theory for diffusive media [40]. Typically, numerical algorithms such as a Monte Carlo or finite-element methods (FEM) are employed to invert the diffusion equation [74]. A priori information (surface topography and the optical properties) and regularization approaches (mainly Tikhonov method [75]) are usually required to solve the ill-posed inverse problem [70, 76]. CT and magnetic resonance imaging (MRI) may provide anatomical information, useful for the surface segmentation and for the source localization, but neither of them gives contrast that is directly related to the tissue optical properties. The surface topography can be obtained also with structured light irradiation at an oblique angle, with the method explained in [74].

In fluorescence tomography the emission takes place at a specific known wavelength, therefore the optical properties of the subject at that wavelength can be measured a priori or during the acquisition, as done for example in fluorescence molecular tomography (FMT, [77]). The intensity of the source is proportional to the absorption coefficient of the fluorophore (at least for low concentrations). This absorption coefficient is itself proportional to the fluorophore concentration in the tissue, that is related to the biological process of interest. In a similar way, bioluminescence tomography (BLT) detects emission on a limited wavelength range (500–565 nm), and the intensity of the bioluminescent source is proportional to the activity of the biological process [70]. In Cerenkov luminescence tomography (CLT), the emission is on a broader range and the correlation between the Cerenkov light distribution and the biological process is neither direct nor linear, because the Cerenkov emission depends on factors as the refractive index of the tissue, the isotope and the source geometry.

### Applications and state of the art

As discussed in the introduction, Cerenkov luminescence imaging is particularly promising to image *β* ^−^ emitters, which are widely used for example in internal radiotherapy but are difficult to visualize with other methods, such as Bremsstrahlung imaging. In addition, CLI is able to provide images in a fast, easy and cost-effective way compared to other methods in nuclear medicine, PET of *β* ^+^-emitters [78] and single photon emission computed tomography (SPECT) of *γ*-emitters [79], and in this respect CLI would allow a larger employment of nuclear medicine probes also in facilities that do not have conventional tomographic systems available. Apart from these uses related to tumor diagnosis and treatment by injection of radionuclides, encouraging applications for CLI are endoscopic and intraoperative ones, as well as dosimetry in external beam therapy.

In this part of the paper, we first describe which are the sources of Cerenkov luminescence of biomedical interest, to underline the potential capabilities of CLI. We then give an overview of the literature on this novel imaging modality, trying to cover all suggested uses up to date.

#### *Biomedical sources of Cerenkov luminescence*

We have seen that Cerenkov luminescence can be produced by charged particles of sufficient energy, and that for electrons and positrons the Cerenkov threshold in tissue is approximately 220 keV. Therefore, in principle any *β*-emitting radioactive source producing decay products with energy above this value can be imaged with CLI. However, different radioisotopes will produce a signal of different intensity (producing between 1–100 Cerenkov photons per decay [32]), and thus will have a different minimum detectable activity level.

The PET radionuclide which has been mostly studied with CLI is certainly ^18^F, as it is widely used in clinical practice. Other imaged tracers are for example ^13^N, ^64^Cu and ^89^Zr. As for *β* ^−^ emitters, images have been obtained of ^131^I, ^90^Y, and ^32^P, to name but a few. A list of the *β*-emitting radioisotopes that have been studied with CLI up to date is reported in Table 4 (decay mode branching ratios and endpoint energies were taken from [80]). As summarized in [73], the number of Cerenkov photons per disintegration produced by some of the radionuclides in Table 4 is ^64^Cu >^18^F >^89^Zr >^124^I >^68^Ga >^90^Y.

**Table 4:** List of *β*-emitting radioisotopes studied with CLI up to date

Radioisotope	Type of decay	Endpoint (keV)	References
^18^F	*β* ^+^ (97%)	633	[1–3, 11, 12, 32, 35, 36, 110, 115]
			[22, 24, 58, 60, 116, 131, 133, 134]
^13^N	*β* ^+^ (100%)	1198	[1, 58]
^64^Cu	*β* ^+^ (18%)	653	[2, 11, 23, 35, 135]
^68^Ga	*β* ^+^ (88%)	1899	[36, 55]
^89^Zr	*β* ^+^ (23%)	2400	[2, 22, 36, 58, 109, 136]
^124^I	*β* ^+^ (12%)	1534	[2, 57, 113]
^32^P	*β* ^−^ (100%)	1710	[54, 137]
^90^Y	*β* ^−^ (99.9%)	2280	[32, 35, 56, 58, 133, 138]
^111^In	AE after EC^a^ (100%)	448	[35, 36]
^131^I	*β* ^−^ (90%), *γ* (82%)	606, 364	[2, 24, 35, 59, 100, 114]
^177^Lu	*β* ^−^ (78.6%)	498	[35, 56]
^198^Au	*β* ^−^ (99%)	961	[107]

^a^AE is Auger electron emission after electron capture (EC)

The potential to image *γ* emitters such as ^∗99*m*^Tc with optical methods has also been investigated [11, 35, 81–83]. However, the energy of the emitted *γ* (140.5 keV) is well below the Cerenkov threshold. Therefore, it is reasonable to assume that the luminescence observed experimentally (for example in [82]) is not due to Cerenkov effect but rather to other physical processes resulting in luminescent emission (e.g., radioluminescence), as suggested in [83, 84]. In addition, the luminescent emission spectrum of ^∗99*m*^Tc has shown to be heavily material dependent, contrasting with the behavior of Cerenkov light [85].

The applicability of CLI also to *α*-emitting radioisotopes has been investigated [2, 86–88]. However, due to their large mass, the threshold kinetic for *α*-particles to produce Cerenkov radiation in tissue is higher than 1500 MeV, while the typically *α*-decay energies are of the order of a few MeV [33, 87]. Also the energy transfered through elastic scattering to the electrons released during tissue ionization is below the Cerenkov threshold for *β*-particles, because these electrons can receive only a small fraction of the *α* energy ($4m_{e}M_{\alpha }/(M_{\alpha }^{2}+m_{e}^{2})$, where *m*_*e*_ and *M*_*α*_ are the rest masses of the electron and *α*, respectively [36]). For these reasons, the strong optical signal measured in [2, 86] has so far been explained as the Cerenkov emission due to daughter nuclei. In addition, the delay in time of this phenomenon decreases the correlation between the Cerenkov light production with the radioisotope distribution [87], thus limiting the applicability of CLI to *α* emitters.

The last category of biomedical particles which can be imaged with CLI are external radiation beams [14, 15, 18, 89–91]. In fact, megavoltage photon beams, as well as electron and proton beams produced with energies of typical clinical applications (6-18 MeV, [92]) can produce Cerenkov light by secondary electron emission. Monte Carlo simulation results by [61] predict high Cerenkov light fluence rates from external radiotherapy beams (300–500 nW cm ^-2^ (Gy s ^-1^) ^-1^ including the effects of absorption by tissue).

#### *Preclinical applications*

The preclinical field is particularly suitable for CLI applications, because the reduced dimensions of the animal allow the Cerenkov light to escape the tissue surface and to be detected. Many uses have been tested, and an overview of the related references is presented here.

**Fig. 10:**
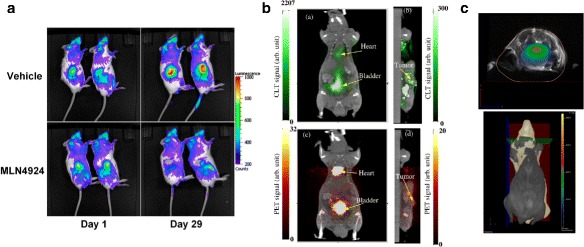
Examples of preclinical CLI and CLT. **a** Tumor treatment monitoring over time in a vehicle group and in a group treated with an anti-tumor agent [93] (with permission). **b** Reconstruction of the activity distribution with Cerenkov luminescence tomography (*green*, *top*) and with PET (*red*, *bottom*) as done by [96] (with permission). **c** Reconstructed msCLT trans-axial slice co-registered with MRI from [54] (with permission). The reconstructed slice position is highlighted in *green* in the bottom part of the figure

**Oncological uses** Preclinical CLI can be used for example to monitor tumor therapy [93, 94] and to monitor radiotherapy with radiolabeled peptides [95]. In the study by [93] (Fig. 10 a), the tumor staging was monitored over time in a mice vehicle group and in a group having the same tumor treated with an anti-tumor agent: CLI data obtained with a PET contrast agent showed the tumor growth over a month in the vehicle group (top row) and its reduction in response to treatment in the other group (bottom row). In another study [95], targeted peptides for the diagnosis and radiotherapy of gastric cancer were radiolabeled with ^131^I and imaged with CLI to assess their tumor binding affinity and antitumor efficacy.

**Cerenkov luminescence tomography** Due to the attenuation of Cerenkov light in tissue, a tomographic acquisition is required to decouple the intensity of the radioactive source from its position at depth in tissue [70]. A few groups have investigated the capability to reconstruct the source position and distribution in tissue with Cerenkov luminescence tomography (CLT). In particular, [96] has shown with phantom and in vivo studies that the source distribution inside objects could be reconstructed from surface measurements using the diffusion equation to model the optical transport and a preconditioned conjugate gradient method to iteratively solve the inverse problem. Figure 10 b shows the result of the reconstruction in tissue (green) superimposed on the anatomical CT image, validated with the PET acquisition (red). The calibrated PET image also allowed the activity quantification, which was not possible from the CLT images. The acquisition was done filtering out short wavelengths to suppress the contribution of Cerenkov photons emitted at the surface of the animal (presumably farther away from the source), and assuming constant optical properties on the detected wavelength range (695–770 nm). This allowed the inversion, but limiting the quality of the reconstruction. A similar study was performed by [97], based on a heterogeneous mouse model rather than on an homogeneous one and using SPECT instead of PET for validation. Also in this case, a bandpass filter was used to collect only long wavelengths (675–775 nm). The problem was further investigated also in [13, 98, 99], and in [100] CLT was used to quantify tumor association of a radiolabeled peptide.

A different approach was used by [54]. This group applied the multi spectral 3D image reconstruction approach called *diffuse luminescence imaging tomography* [74] to obtain a 3D image of the Cerenkov light distribution, and named this technique *multi spectral diffuse Cerenkov luminescence tomography* (msCLT). As the name suggests, this approach combines the information at different wavelengths obtained by images acquired using a number of narrow bandpass filters. Therefore, this method relies on a set of 2D planar images acquired from the same point of view, rather than using multiple projections. The result of the reconstruction is shown in Fig. 10 c for a brain trans-axial slice. The reconstructed Cerenkov light distribution is superimposed on the magnetic resonance anatomical image for localization, and the slice shown is highlighted in green in the bottom part of the figure.

**Cerenkov luminescence for internal excitation** Another interesting application is the release at depth in tissue of excitation light to fluorophores or nanoparticles [11, 12, 101–105]. Applications falling in this category are often referred to as *Cerenkov-excited luminescence* applications [92]. This type of use of Cerenkov light has the potential to increase the light output in two ways: by overcoming the tissue penetration limit of external excitation lamps required for fluorescence imaging, and by simultaneously shifting the Cerenkov spectrum peak towards longer wavelengths (typically *λ*=560−800 nm). Wavelengths in the so-called near-infrared (NIR) window (also known as optical window or therapeutic window, *λ*=650−1350 nm) are in fact more penetrating (Fig. 6). To this purpose, in [22] it has been shown that targeted and activatable fluorescent probes excited by Cerenkov luminescence can be created (coupling ^18^F-FDG to quantum dots), thus allowing to image two molecular information: metabolic activity through ^18^F-FDG and integrin expression through the quantum dot excited by the ^18^F-FDG. In [106], it has been shown that PET probes such as ^18^F-FDG can also be used as a source of light for photoactivation of caged luciferin in a breast cancer animal model expressing luciferase. In another study, radioluminescent Au nanocages were developed with the aid of CLI visualization [107]. To enhance the CLI signal, in [11] PET tracers were coupled to quantum dots to improve optical imaging in biological systems. Another group has suggested using europium oxide nanoparticles excited by the Cerenkov luminescence produced by radiopharmaceuticals to increase sensitivity in a tomographic setting [108].

**Other preclinical CLI applications** Potential uses of preclinical CLI which have been evaluated are also intraoperative imaging of radionuclides [109], surgical resection [110], endoscopic imaging for intraoperative surgical navigation [111], study of gene expression [57, 112–114], dynamic imaging [115], interscapular brown adipose tissue visualization [116], quantitive tissue oxygenation assessment [117], and negative contrast imaging of blood vessel attenuation of Cerenkov light to image vasculature [55]. In [118], Cerenkov-specific contrast agents for pH detection have been developed, while in [119] antibody-based PET radiotracers have been evaluated via CLI.

#### *External beam radiotherapy*

Work has been done also to investigate CLI uses in external beam radiotherapy. The study of [89] has shown that electron beams produce CL which can excite fluorescent agents for dual therapy (from the external beam and from local phototherapy), and projection imaging of photon beams using Cerenkov-excited fluorescence has been evaluated in [14]. CLI has been proposed also as a tool to monitor the efficacy of external beam radiotherapy [15], suggesting time-gated acquisition to avoid the issues coming from unwanted ambient light [120]. The use of CLI has been proposed also for beam quality assurance and for dose calibration [121, 122]. Two more recent studies showed how CLI can visualize radiation therapy in real time during breast radiation therapy [91], and used beams produced by medical linear accelerators to induce Cerenkov luminescence in tissue phantoms, naming this technique CELSI, for Cerenkov-excited luminescence scanned imaging [62]. Another study used CLI for patient positioning and movement tracking [18]. The use of the Cerenkov light generated in plastic optical fibers for proton [26] and photon [123] therapy dosimetry has also been considered. Finally, simulation results have analyzed theoretically the physics of Cerenkov emission during proton therapy [16], and the correlation between the captured Cerenkov light and the expected dose for x-ray photons, electrons, and protons [122].

#### *Quantitative CLI*

**Fig. 11:**
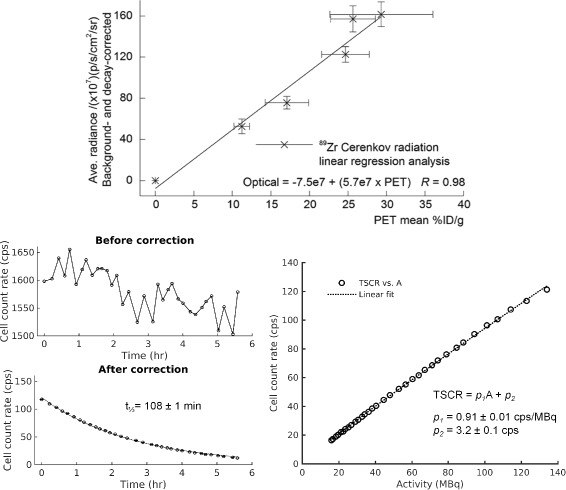
Examples of quantitative CLI. *Top*: Quantitative analysis of CLI and PET signals [2] (with permission). *Bottom left*: Decay in time of the count rate due to ^18^F measured by the PDPC, before and after data correction [27]. *Bottom right*: Linearity of the Cerenkov count rate in the PDPC with the ^18^F activity level, adapted from [69]

Some groups have also investigated the quantitative capabilities of CLI. This was done for example by correlating quantitatively the CLI signal with either the PET or SPECT signal [94]. Other studies have shown that the CLI signal is linear with dose in external therapy [15], or with activity in radiopharmaceutical imaging [124]. An example of the correlation of the CLI signal with the PET signal (*x*-axis, expressed in mean percent injected activity per gram) is shown in Fig. 11 (top). The correlation between the two signals is high (*R* ^2^=0.98), and the fit equation y(x) calibrates the signal measured with CLI with that of PET. Since PET can be calibrated to quantify the functional information, by means of this cross-calibration it is possible to relate the CLI measured quantity with the source activity distribution, thus fulfilling both requirements of functional imaging (imaging and quantification of the activity distribution). An overview of the published literature on the correlation of CLI and PET can be found in [67].

One study has investigated the quantitative capabilities of digital silicon photomultipliers (dSiPMs) for CLI [69]. In fact, dSiPMs provide directly the number of detected photons and might therefore allow an easier quantitative reconstruction of the activity with respect to CCDs (which instead require some sort of calibration to recover the number of detected photons). This preliminary feasibility study showed that, with a sequential activation of the cells of the dSiPM and with proper data correction, the detector responded dynamically to different activity levels and source depths in tissue. In particular, the ^18^F half life could be obtained from the experimental data with very high precision. Figure 11 (bottom left) shows the effect of the data correction algorithm on the ^18^F decay curve in time (from [27]), Figure 11 (bottom right) shows the linearity of the Cerenkov count rate in the PDPC with the ^18^F activity level, after data correction (adapted from [69]).

#### *Simulation studies*

A few groups have compared CLI results with Monte Carlo simulations that predicted the light production in different conditions. In fact, as we have seen the Cerenkov light production depends on several factors. Therefore, it is not trivial to determine the Cerenkov light distribution produced by a given activity distribution and the intensity of the detectable signal in a certain experimental condition.

**Fig. 12:**
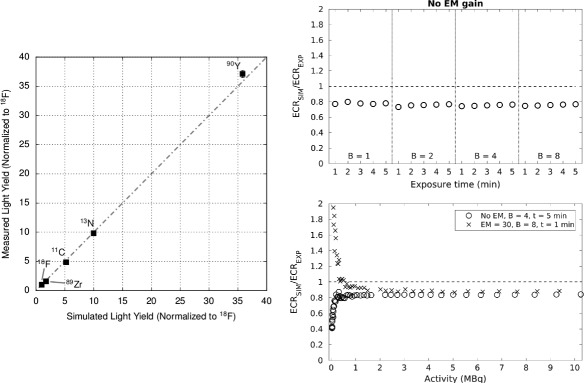
*Left*: Relative comparison of measured and simulated Cerenkov light yield for a few radionuclides in water [58] (with permission). *Right*: Simulated over measured Cerenkov count rate with different acquisition settings (top) and as a function of activity, for two combinations of acquisition settings (bottom); both figures were adapted from [125]

In particular, [32, 58] have developed a code to determine the Cerenkov light production in water and tissue for many isotopes. The light yield predicted by their Monte Carlo code in [58] is shown in Fig. 12 (left), plotted versus the light yield they measured experimentally for some isotopes. As indicated in the graph axis, only a *relative* comparison of the simulated and measured light yield could be done (both quantities are normalized to the ^18^F light yield) due to a constant factor two-discrepancy between simulated and experimental results reported by the authors. In [58], the group has also studied the dependence of the Cerenkov light production on factors like the index of refraction and the geometry of the experimental setup.

A similar study has been performed also by [36]. The results of this group have shown to be consistent with those of [32] and a set of corrections of the simulated predictions to match the experimental results have been proposed.

In [27, 125], analogous simulations in Geant4 have been performed and compared with experimental measurements. In this case, a custom-built CCD-based imaging system featuring an EMCCD was used rather than the IVIS. The geometrical light collection efficiency of the lens was determined experimentally, and inserted in the Monte Carlo simulation together with the information available on the detector spectral response. This allowed an *absolute* quantitative comparison of the number of detected photons predicted by the simulation and measured experimentally. Preliminary results using ^18^F and ^90^Y in water solutions suggested that the Monte Carlo is able to predict the measured Cerenkov photon counts with only a 20% underestimation of the true values. As shown in Fig. 12 (right) (adapted from [125]), this constant offset was reproducible among different acquisition settings (e.g., varying the pixel binning factor and exposure time, top) and different activity levels (bottom), provided that the activity was above the minimum radioisotope activity level that can be detected with CLI. Additional measurements with tissue samples and emission filters to separate Cerenkov spectral components are being carried out to confirm these preliminary results.

Other groups have done simulation studies with different goals. In particular, in [15] CLI measurements have been compared with Monte Carlo predictions of superficial dose distribution in external radio therapy, and a hybrid Monte Carlo tool has been developed [126] that has been used for a breast study [127] and for small animal imaging [128].

#### *Clinical applications*

**Fig. 13:**
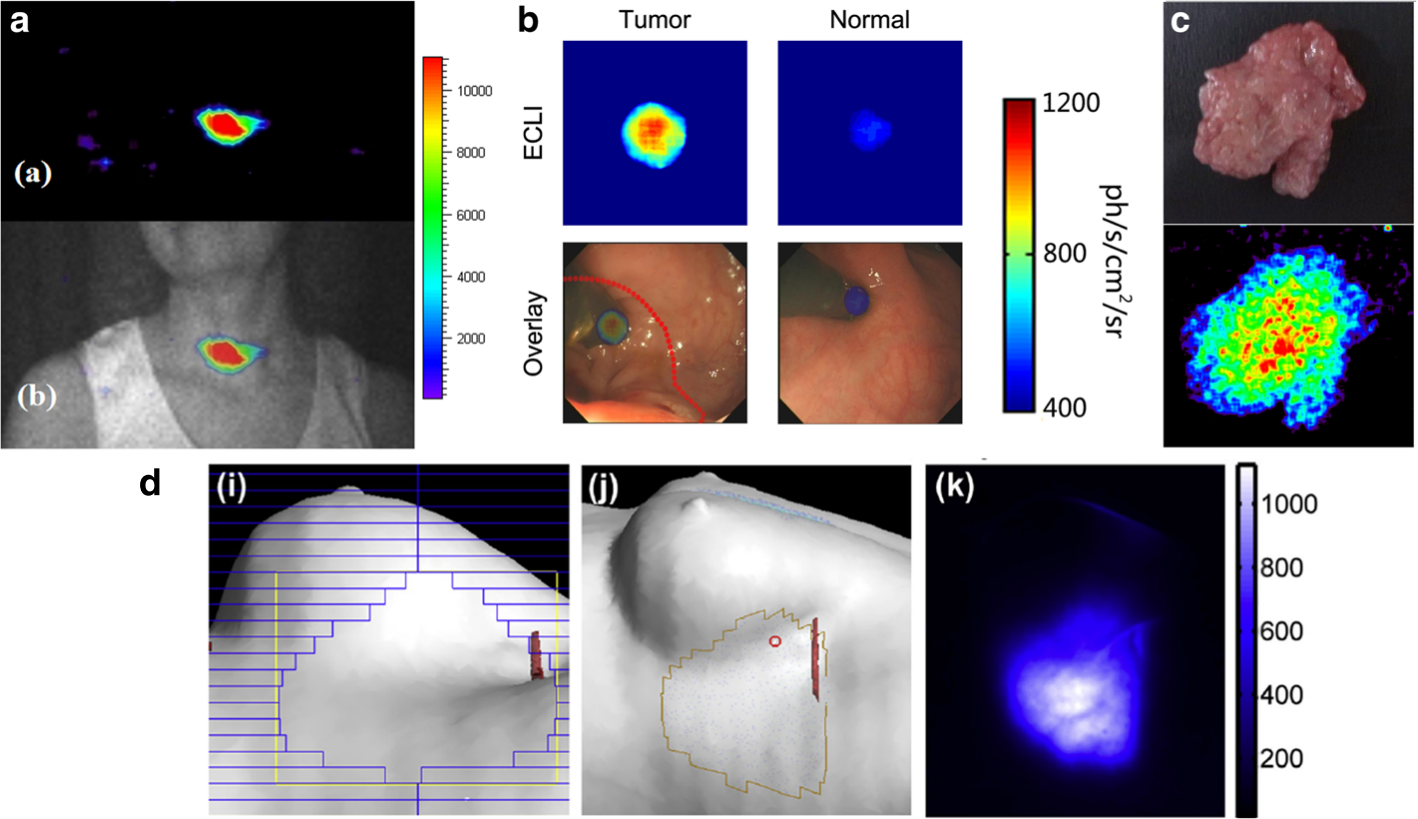
Examples of clinical CLI. **a** First human CLI image of a thyroid suffering from hyperthyroidism treated with 550 MBq of ^131^I-iodide, 24 h after administration [59] (with permission). **b** CLI endoscopy (ECLI) of cancerous (*left*) and normal tissue (*right*), by [20] (with permission). **c** *Ex-vivo* CLI of human brain tumor removed during neurosurgery, by [130] (with permission). **d** Real-time CLI of human breast external radiation therapy, by [91] (with permission): beam’s eye view of treatment (*left*), corresponding skin projection (*center*) and Cerenkov emission image (*right*)

Clinical images of ^131^I-iodide metabolic radiotheraphy of a diseased thyroid (Fig. 13 a, [59]) and of ^18^F-FDG positive lymph nodes [60] have been acquired successfully. In the clinical field, CLI could be advantageous also during endoscopy (by exploiting the natural body cavities) and useful in intraoperative practice, to detect and highlight diseased tissues and to guide surgical resection [19, 21, 109, 129]. The first clinical endoscopic CLI imaging system has been developed for human studies, and preliminary images of diseased and healthy tissue are shown in Fig. 13 b by [20]. A successful resection of a sentinel lymph node has been shown by [110], while in [130] ex vivo CLI of a human brain tumor specimen removed during neurosurgery has been shown (Fig. 13 c). A CLI intra-operative imaging device has been developed by LightPoint Medical Inc., and the first clinical trials are ongoing to demonstrate its utility to assess tumor margins during surgery [67]. As for external beam radiotheraphy, an example of the real-time imaging of a breast treatment is shown Fig. 13 d by [91]: the beam’s eye view of the treatment is shown on the left, together with the corresponding skin projection (center) and Cerenkov emission image (right).

#### *Further perspectives*

One important aspect to be considered to fully exploit the potential of CLI as a stand-alone imaging modality is to understand its quantitative capabilities. Taking for example the functional imaging of a radioactive distribution, *quantitative CLI* means that the measured quantity (a signal proportional to the Cerenkov radiance emitted at the tissue surface) is quantitatively and spatially correlated to the activity distribution at depth in tissue (as mentioned earlier, due to the attenuation of optical light in tissue, this can potentially be achieved only with a tomographic acquisition).

In PET, the requirements of quantitative and spatial correlation of the measured signal to the activity distribution are fulfilled by combining the PET functional information with the morphological information obtained with computed tomography (CT). The CT measurement provides the source localization and the correction for the attenuation of the detected PET signal. In addition, the quantitative information is obtained by calibrating the response of the PET imaging system with a reference standard, e.g., a phantom uniformly filled with a radioactive solution.

With respect to PET, the reconstruction in CLT is complicated by the absence of a tissue equivalent material able to mimic the tissue interactions with visible light over the entire Cerenkov spectrum and by the lack of a reference standard (as is water for PET) to calibrate the detector response in terms of emitted radiance at the tissue surface. In addition, the optical properties of the various tissues, that regulate the light transport between production and emission at the tissue surface (equivalent to what in PET is corrected with the CT attenuation correction), are often not known, and available data are restricted to a few wavelengths. Also with respect to conventional optical tomography (bioluminescence and fluorescence tomography), the broad-band nature of the Cerenkov emission and the complex correlation between the distribution of the emitted Cerenkov light and the activity distribution complicate the CLT reconstruction.

For all these reasons, up to date a complete quantitative reconstruction of the activity distribution from a CLT acquisition has not been demonstrated, unless by means of a cross-calibration with PET or SPECT (see for example [96]). In principle, for a tissue with known optical properties, the inverse problem in CLT could be solved with the following steps:

1. calibration of the response of the imaging system to obtain the surface radiance;
2. reconstruction of the Cerenkov light distribution at production at depth in tissue from the surface radiance;
3. reconstruction of the isotope distribution from the Cerenkov light distribution;
4. localization of the activity distribution.

Supposing that the first two steps of this algorithm can be borrowed by conventional optical tomography, that the optical properties of the tissue can be somewhat measured and that anatomical information is available, one possible solution to correlate quantitatively the Cerenkov light production distribution to the radiotracer distribution is to use Monte Carlo simulations to predict the Cerenkov light output of a given distribution, as those in [27, 32, 36, 58]. However, the feasibility of this method in practice still needs to be fully proven and would be a very interesting subject for future studies.

## Conclusions

Cerenkov luminescence imaging (CLI) is a simple, fast and cost-effective tool to image radioisotopes and particle beams. Yet, CLI suffers from all the drawbacks of optical imaging (reduced penetration depth and complex transport in tissue), with additional limitations due to the broader emission spectrum and to the non-linear correlation with the distribution of the primary source responsible of the Cerenkov emission. However, the potentialities of this technique in the preclinical, but also clinical field, justify a growing interest toward CLI. In fact, this imaging modality can be used to study radiotracers and radiopharmaceuticals employed for oncological uses, also in endoscopic and intraoperative contexts. It can be useful also for dosimetry and other purposes in photon, electron and proton beam therapy. The Cerenkov signal can be enhanced if used to excite fluorescent agents, having potential usability also for photoactivation and phototherapy. Nevertheless, further research is needed in this field to move from a qualitative to a true quantitative molecular imaging technique.

## Endnote

^1^ It should be noted that recently a technique termed radioluminescence imaging (RLI) has been explored [84, 131], but this method uses the luminescence produced by radioactive sources in scintillating crystals rather than in tissue.

## References

[1] Robertson R, Germanos M, Li C, Mitchell G, Cherry S, Silva M (2009). Optical imaging of Cerenkov light generation from positron-emitting radiotracers. Phys Med Biol.

[2] Ruggiero A, Holland J, Lewis J, Grimm J (2010). Cerenkov luminescence imaging of medical isotopes. J Nucl Med.

[3] Spinelli A, D’Ambrosio D, Calderan L, Marengo M, Sbarbati A, Boschi F (2010). Cerenkov radiation allows in vivo optical imaging of positron emitting radiotracers. Phys Med Biol.

[4] Shen S, DeNardo G, DeNardo S (1994). Quantitative Bremsstrahlung imaging of Yttrium-90 using a Wiener filter. Med Phys.

[5] Walrand S, Flux G, Konijnenberg M, Valkema R, Krenning E, Lhommel R, Pauwels S, Jamar F (2011). Dosimetry of Yttrium-labelled radiopharmaceuticals for internal therapy: 86Y or 90Y imaging?. Eur J Nuclear Med Mol Imaging.

[6] Minarik D, Gleisner K, Ljungberg M (2008). Evaluation of quantitative 90Y SPECT based on experimental phantom studies. Phys Med Biol.

[7] Minarik D, Ljungberg M, Segars P (2009). Evaluation of quantitative planar 90Y Bremsstrahlung whole-body imaging. Phys Med Biol.

[8] Padia S, Alessio A, Kwan S, Lewis D, Vaidya S, Minoshima S (2013). Comparison of Positron Emission Tomography and Bremsstrahlung imaging to detect particle distribution in patients undergoing Yttrium-90 radioembolization for large hepatocellular carcinomas or associated portal vein thrombosis. J Vasc Interv Radiol.

[9] Clarke L, Cullom S, Shaw R, Reece C, Penney B, King M, Silbiger M (1992). Bremsstrahlung imaging using the gamma camera: factors affecting attenuation. J Nucl Med.

[10] Kappadath S (2010). SU-GG-I-163: A scatter correction algorithm for quantitative Yttrium-90 SPECT imaging. Med Phys.

[11] Dothager R, Goiffon R, Jackson E, Harpstrite S, Piwnica-Worms D (2010). Cerenkov radiation energy transfer (CRET) imaging: a novel method for optical imaging of PET isotopes in biological systems. PloS ONE.

[12] Lewis M, Kodibagkar V, Öz O, Mason R (2010). On the potential for molecular imaging with Cerenkov luminescence. Opt Lett.

[13] Zhong J, Qin C, Yang X, Zhu S, Zhang X, Tian J (2011). Cerenkov luminescence tomography for in vivo radiopharmaceutical imaging. J Biomed Imaging.

[14] Glaser AK, Davis SC, McClatchy DM, Zhang R, Pogue BW, Gladstone DJ (2013). Projection imaging of photon beams by the čerenkov effect. Med Phys.

[15] Zhang R, Fox C, Glaser A, Gladstone D, Pogue B (2013). Superficial dosimetry imaging of čerenkov emission in electron beam radiotherapy of phantoms. Phys Med Biol.

[16] Helo Y, Kacperek A, Rosenberg I, Royle G, Gibson A (2014). The physics of Cerenkov light production during proton therapy. Phys Med Biol.

[17] Yamamoto S, Toshito T, Fujii K, Morishita Y, Okumura S, Komori M (2014). High resolution Cerenkov light imaging of induced positron distribution in proton therapy. Med Phys.

[18] Zhang R, Andreozzi J, Gladstone D, Hitchcock W, Glaser A, Jiang S, Pogue B, Jarvis L (2014). Cherenkoscopy based patient positioning validation and movement tracking during post-lumpectomy whole breast radiation therapy. Phys Med Biol.

[19] Kothapalli S, Liu H, Liao J, Cheng Z, Gambhir S (2012). Endoscopic imaging of Cerenkov luminescence. Biomed Opt Express.

[20] Hu H, Cao X, Kang F, Wang M, Lin Y, Liu M, Li S, Yao L, Liang J, Liang J (2015). Feasibility study of novel endoscopic Cerenkov luminescence imaging system in detecting and quantifying gastrointestinal disease: first human results. Eur Radiol.

[21] Liu H, Carpenter C, Jiang H, Pratx G, Sun C, Buchin M, Gambhir S, Xing L, Cheng Z (2012). Intraoperative imaging of tumors using Cerenkov luminescence endoscopy: a feasibility experimental study. J Nucl Med.

[22] Thorek D, Ogirala A, Beattie B, Grimm J (2013). Quantitative imaging of disease signatures through radioactive decay signal conversion. Nat Med.

[23] Li J, Dobrucki L, Marjanovic M, Chaney E, Suslick K, Boppart S (2015). Enhancement and wavelength-shifted emission of Cerenkov luminescence using multifunctional microspheres. Phys Med Biol.

[24] Shimamoto M, Gotoh K, Hasegawa K, Kojima A (2016). Hybrid light imaging using Cerenkov luminescence and liquid scintillation for preclinical optical imaging in vivo. Mol Imaging Biol.

[25] Korpar S, Dolenec R, Križan P, Pestotnik R, Stanovnik A (2011). Study of TOF PET using Cherenkov light. Nuclear Instruments Methods Phys Res Section A Accelerators Spectrometers Detectors Assoc Equipment.

[26] Jang K, Yoo W, Shin S, Shin D, Lee B (2012). Fiber-optic Cerenkov radiation sensor for proton therapy dosimetry. Opt Express.

[27] Ciarrocchi E. Cerenkov luminescence imaging: a study of its quantitative capabilities. PhD thesis, University of Pisa, Department of Physics 2016.

[28] L’Annunziata M (2012). Handbook of radioactivity analysis.

[29] Jelley J (1958). Cherenkov radiation and its applications.

[30] Fernow R (1986). Introduction to experimental particle physics.

[31] Green D (2000). The physics of particle detectors.

[32] Mitchell G, Gill R, Boucher D, Li C, Cherry S (2011). In vivo Cerenkov luminescence imaging: a new tool for molecular imaging. Phil Trans R Soc A Math Phys Eng Sci.

[33] Das S, Thorek D, Grimm J (2014). Cerenkov imaging. Adv Cancer Res.

[34] Jelley J (1955). Cerenkov radiation and its applications. Br J Appl Phys.

[35] Liu H, Ren G, Miao Z, Zhang X, Tang X, Han P, Gambhir S, Cheng Z (2010). Molecular optical imaging with radioactive probes. PLoS One.

[36] Beattie B, Thorek D, Schmidtlein C, Pentlow K, Humm J, Hielscher A (2012). Quantitative modeling of Cerenkov light production efficiency from medical radionuclides. PloS ONE.

[37] Andersson-Engels S, af Klinteberg C, Svanberg K, Svanberg S (1997). In vivo fluorescence imaging for tissue diagnostics. Phys Med Biol.

[38] Chin P, Welling M, Meskers S, Olmos R, Tanke H, van Leeuwen F (2013). Optical imaging as an expansion of nuclear medicine: Cerenkov-based luminescence vs fluorescence-based luminescence. Eur J of Nucl Med Mol Imaging.

[39] Bremer C, Ntziachristos V, Weissleder R (2003). Optical-based molecular imaging: contrast agents and potential medical applications. Eur Radiol.

[40] Wang L, Wu H (2007). Biomedical optics: principles and imaging.

[41] Cheong W, Prahl S, Welch A (1990). A review of the optical properties of biological tissues. iEEE J Quantum Electron.

[42] Tuchin V (2007). Tissue optics: light scattering methods and instruments for medical diagnosis.

[43] Jacques S (2013). Optical properties of biological tissues: a review. Phys Med Biol.

[44] The Oregon Medical Laser Center Website. http://omlc.org/spectra/hemoglobin/. Accessed on 17 Feb 2017.

[45] Hale G, Querry M (1973). Optical constants of water in the 200-nm to 200- *μ*m wavelength region. Appl Opt.

[46] Lister T, Wright P, Chappell P (2012). Optical properties of human skin. J Biomed Opt.

[47] Chan E, Sorg B, Protsenko D, O’Neil M, Motamedi M, Welch A (1996). Effects of compression on soft tissue optical properties. IEEE J Selected Topics Quantum Electron.

[48] Marquez G, Wang L, Lin S, Schwartz J, Thomsen S (1998). Anisotropy in the absorption and scattering spectra of chicken breast tissue. Appl Opt.

[49] Huang Z, McWilliams A, Lui H, McLean DI, Lam S, Zeng H (2003). Near-infrared raman spectroscopy for optical diagnosis of lung cancer. Intl J Cancer.

[50] Ntziachristos V (2010). Going deeper than microscopy: the optical imaging frontier in biology. Nat Methods.

[51] Jackson J (1975). Classical Electrodynamics.

[52] Bolin FP, Preuss LE, Taylor RC, Ference RJ (1989). Refractive index of some mammalian tissues using a fiber optic cladding method. Appl Opt.

[53] Boschi F, Calderan L, D’Ambrosio D, Marengo M, Fenzi A, Calandrino R, Sbarbati A, Spinelli AE (2011). In vivo 18F-FDG tumour uptake measurements in small animals using Cerenkov radiation. Eur J of Nucl Med Mol Imaging.

[54] Spinelli A, Kuo C, Rice B, Calandrino R, Marzola P, Sbarbati A, Boschi F (2011). Multispectral Cerenkov luminescence tomography for small animal optical imaging. Opt Express.

[55] Steinberg J, Raju A, Chandrasekharan P, Yang C, Khoo K, Abastado J, Robins E, Townsend D (2014). Negative contrast Cerenkov luminescence imaging of blood vessels in a tumor mouse model using [68 GA] gallium chloride. EJNMMI Res.

[56] Balkin E, Kenoyer A, Orozco J, Hernandez A, Shadman M, Fisher D, Green D, Hylarides M, Press O, Wilbur D (2014). In vivo localization of 90Y and 177Lu radioimmunoconjugates using Cerenkov luminescence imaging in a disseminated murine leukemia model. Cancer Res.

[57] Wolfs E, Holvoet B, Gijsbers R, Casteels C, Roberts S, Struys T, Maris M, Ibrahimi A, Debyser Z, Van Laere K (2014). Optimization of multimodal imaging of mesenchymal stem cells using the human sodium iodide symporter for PET and Cerenkov luminescence imaging. PloS one.

[58] Gill R, Mitchell G, Cherry S (2015). Computed Cerenkov luminescence yields for radionuclides used in biology and medicine. Phys Med Biol.

[59] Spinelli A, Ferdeghini M, Cavedon C, Zivelonghi E, Calandrino R, Fenzi A, Sbarbati A, Boschi F (2013). First human cerenkography. J Biomed Opt.

[60] Thorek D, Riedl C, Grimm J (2014). Clinical Cerenkov luminescence imaging of 18F-FDG. J Nucl Med.

[61] Glaser A, Zhang R, Andreozzi J, Gladstone D, Pogue B (2015). Cherenkov radiation fluence estimates in tissue for molecular imaging and therapy applications. Phys Med Biol.

[62] Zhang R, D’souza A, Gunn J, Esipova T, Vinogradov S, Glaser A, Jarvis L, Gladstone D, Pogue B (2015). Cherenkov-excited luminescence scanned imaging. Opt Lett.

[63] Leblond F, Davis S, Valdés P, Pogue B (2010). Pre-clinical whole-body fluorescence imaging: Review of instruments, methods and applications. J Photochem Photobiol B Biol.

[64] Zhang L, Neves L, Lundeen J, Walmsley I (2009). A characterization of the single-photon sensitivity of an electron multiplying charge-coupled device. J Phys B Atomic Mol Optical Phys.

[65] Robbins M, Hadwen B (2003). The noise performance of electron multiplying charge-coupled devices. Electron Devices IEEE Trans.

[66] Holst G, Lomheim T (2007). CMOS/CCD sensors and camera systems.

[67] Grootendorst M, Cariati M, Kothari A, Tuch D, Purushotham A (2016). Cerenkov luminescence imaging (cli) for image-guided cancer surgery. Clin Transl Imaging.

[68] Spinelli A, Boschi F (2012). Optimizing in vivo small animal Cerenkov luminescence imaging. J Biomed Opt.

[69] Ciarrocchi E, Belcari N, Del Guerra A, Cherry S, Lehnert A, Hunter W, McDougald W, Miyaoka R, Kinahan P (2015). Cherenkov luminescence measurements with digital Silicon Photomultipliers: a feasibility study. EJNMMI Phys.

[70] Darne C, Lu Y, Sevick-Muraca E (2013). Small animal fluorescence and bioluminescence tomography: a review of approaches, algorithms and technology update. Phys Med Biol.

[71] Orosco R, Tsien R, Nguyen Q (2013). Fluorescence imaging in surgery. IEEE Rev Biomed Eng.

[72] Troy T, Jekic-McMullen D, Sambucetti L, Rice B (2004). Quantitative comparison of the sensitivity of detection of fluorescent and bioluminescent reporters in animal models. Mol Imaging.

[73] Thorek D, Robertson R, Bacchus W, Hahn J, Rothberg J, Beattie B, Grimm J (2012). Cerenkov imaging - a new modality for molecular imaging. Am J Nucl Med Mol Imaging.

[74] Kuo C, Coquoz O, Troy T, Xu H, Rice BW (2007). Three-dimensional reconstruction of in vivo bioluminescent sources based on multispectral imaging. J Biomed Opt.

[75] Tikhonov A. Solution of incorrectly formulated problems and the regularization method. In: Soviet Math. Dokl: 1963. p. 1035–8.

[76] Dehghani H, Eames M, Yalavarthy P, Davis S, Srinivasan S, Carpenter C, Pogue B, Paulsen K (2009). Near infrared optical tomography using NIRFAST: Algorithm for numerical model and image reconstruction. Commun Numer Methods Eng.

[77] Ntziachristos V, Tung C, Bremer C, Weissleder R (2002). Fluorescence molecular tomography resolves protease activity in vivo. Nat Med.

[78] Del Guerra A, Belcari N, Bisogni M (2016). Positron Emission Tomography: Its 65 years. Rivista Del Nuovo Cimento.

[79] Mariani G, Bruselli L, Kuwert T, Kim EE, Flotats A, Israel O, Dondi M, Watanabe N (2010). A review on the clinical uses of SPECT/CT. Eur J Nucl Med Mol Imaging.

[80] Ma X, Wang J, Cheng Z (2014). Cerenkov radiation: a multi-functional approach for biological sciences. Front Phys.

[81] Boschi F, Spinelli A, D’Ambrosio D, Calderan L, Marengo M, Sbarbati A (2009). Combined optical and single photon emission imaging: preliminary results. Phys Med Biol.

[82] Spinelli A, Meo S, Calandrino R, Sbarbati A, Boschi F (2011). Optical imaging of Tc-99m–based tracers: in vitro and in vivo results. J Biomed Opt.

[83] Kondakov A, Gubskiy I, Znamenskiy I, Chekhonin V (2014). Possibilities of optical imaging of the Tc99m-based radiopharmaceuticals. J Biomed Opt.

[84] Spinelli A, Boschi F (2015). Novel biomedical applications of Cerenkov radiation and radioluminescence imaging. Phys Med.

[85] Pagliazzi M, Boschi F, Spinelli A (2014). Imaging of luminescence induced by beta and gamma emitters in conventional non-scintillating materials. RSC Adv.

[86] Boschi F, Meo S, Rossi P, Calandrino R, Sbarbati A, Spinelli A (2011). Optical imaging of alpha emitters: simulations, phantom, and in vivo results. J Biomed Opt.

[87] Ackerman N, Graves E (2012). The potential for Cerenkov luminescence imaging of alpha-emitting radionuclides. Phys Med Biol.

[88] Wood V, Ackerman N (2016). Cherenkov light production from the *α*-emitting decay chains of 223 Ra, 212 Pb, and 149 Tb for Cherenkov luminescence imaging. Appl Radiat Isot.

[89] Axelsson J, Davis S, Gladstone D, Pogue B (2011). Cerenkov emission induced by external beam radiation stimulates molecular fluorescence. Med Phys.

[90] Zhang R, Gladstone D, Jarvis L, Strawbridge R, Hoopes PJ, Friedman O, Glaser A, Pogue B (2013). Real-time in vivo cherenkoscopy imaging during external beam radiation therapy. J Biomed Opt.

[91] Jarvis L, Zhang R, Gladstone D, Jiang S, Hitchcock W, Friedman O, Glaser A, Jermyn M, Pogue B (2014). Cherenkov video imaging allows for the first visualization of radiation therapy in real time. Int J Radiat Oncol Biol Phys.

[92] Tanha K, Pashazadeh A, Pogue B (2015). Review of biomedical čerenkov luminescence imaging applications. Biomed Opt Express.

[93] Robertson R, Germanos M, Manfredi M, Smith P, Silva M (2011). Multimodal imaging with 18F-FDG PET and Cerenkov luminescence imaging after MLN4924 treatment in a human lymphoma xenograft model. J Nucl Med.

[94] Xu Y, Chang E, Liu H, Jiang H, Gambhir SS, Cheng Z (2012). Proof-of-concept study of monitoring cancer drug therapy with Cerenkov luminescence imaging. J Nucl Med.

[95] Zhang J, Hu H, Liang S, Yin J, Hui X, Hu S, He M, Wang J, Wang B, Nie Y (2013). Targeted radiotherapy with tumor vascular homing trimeric GEBP11 peptide evaluated by multimodality imaging for gastric cancer. J Control Release.

[96] Li C, Mitchell G, Cherry S (2010). Cerenkov luminescence tomography for small-animal imaging. Opt Lett.

[97] Hu Z, Liang J, Yang W, Fan W, Li C, Ma X, Chen X, Ma X, Li X, Qu X (2010). Experimental Cerenkov luminescence tomography of the mouse model with SPECT imaging validation. Opt Express.

[98] Zhong J, Tian J, Yang X, Qin C (2011). Whole-body Cerenkov luminescence tomography with the finite element sp3 method. Annal Biomed Eng.

[99] Zhong J, Qin C, Yang X, Chen Z, Yang X, Tian J (2012). Fast-specific tomography imaging via Cerenkov emission. Mol Imaging Biol.

[100] Hu Z, Yang W, Ma X, Ma W, Qu X, Liang J, Wang J, Tian J (2013). Cerenkov luminescence tomography of aminopeptidase N (APN/CD13) expression in mice bearing ht1080 tumors. Mol Imaging.

[101] Liu H, Zhang X, Xing B, Han P, Gambhir S, Cheng Z (2010). Radiation-luminescence-excited quantum dots for in vivo multiplexed optical imaging. Small.

[102] Xu Y, Liu H, Cheng Z (2011). Harnessing the power of radionuclides for optical imaging: Cerenkov luminescence imaging. J Nucl Med.

[103] Boschi F, Spinelli A (2012). Quantum dots excitation using pure beta minus radioisotopes emitting Cerenkov radiation. RSC Adv.

[104] Kotagiri N, Niedzwiedzki D, Ohara K, Achilefu S (2013). Activatable probes based on distance-dependent luminescence associated with Cerenkov radiation. Angew Chem Int Ed.

[105] Kotagiri N, Sudlow G, Akers W, Achilefu S (2015). Breaking the Depth dependency of phototherapy with Cerenkov radiation and low-radiance-responsive nanophotosensitizers. Nat Nanotechnol.

[106] Ran C, Zhang Z, Hooker J, Moore A (2012). In vivo photoactivation without “light”: use of Cherenkov radiation to overcome the penetration limit of light. Mol Imaging Biol.

[107] Wang Y, Liu Y, Luehmann H, Xia X, Wan D, Cutler C, Xia Y (2013). Radioluminescent gold nanocages with controlled radioactivity for real-time in vivo imaging. Nano Lett.

[108] Hu Z, Zhao M, Qu Y, Zhang X, Zhang M, Liu M, Guo H, Zhang Z, Wang J, Yang W (2017). In vivo 3-dimensional radiopharmaceutical-excited fluorescence tomography. J Nucl Med.

[109] Holland J, Normand G, Ruggiero A, Lewis J, Grimm J (2011). Intraoperative imaging of Positron Emission Tomographic radiotracers using Cerenkov luminescence emissions. Mol Imaging.

[110] Thorek D, Abou D, Beattie B, Bartlett R, Huang R, Zanzonico P, Grimm J (2012). Positron lymphography: multimodal, high-resolution, dynamic mapping and resection of lymph nodes after intradermal injection of 18F-FDG. J Nucl Med.

[111] Song T, Liu X, Qu Y, Liu H, Bao C, Leng C, Hu Z, Wang K, Tian J (2015). A novel endoscopic Cerenkov luminescence imaging system for intraoperative surgical navigation. Mol Imaging.

[112] Liu H, Ren G, Liu S, Zhang X, Chen L, Han P, Cheng Z (2010). Optical imaging of reporter gene expression using a positron-emission-tomography probe. J Biomed Opt.

[113] Jeong S, Hwang M, Kim J, Kang S, Park J, Yoo J, Ha J, Lee S, Ahn B, Lee J (2011). Combined Cerenkov luminescence and nuclear imaging of radioiodine in the thyroid gland and thyroid cancer cells expressing sodium iodide symporter: initial feasibility study. Endocr J.

[114] Yang W, Qin W, Hu Z, Suo Y, Zhao R, Ma X, Ma W, Wang T, Liang J, Tian J (2012). Comparison of Cerenkov Luminescence Imaging (CLI) and gamma camera imaging for visualization of let-7 expression in lung adenocarcinoma A549 cells. Nucl Med Biol.

[115] Spinelli A, Boschi F (2011). Unsupervised analysis of small animal dynamic Cerenkov luminescence imaging. J Biomed Opt.

[116] Zhang X, Kuo C, Moore A, Ran C (2013). In vivo optical imaging of interscapular brown adipose tissue with 18F-FDG via Cerenkov luminescence imaging. PloS ONE.

[117] Axelsson J, Glaser A, Gladstone D, Pogue B (2012). Quantitative Cherenkov emission spectroscopy for tissue oxygenation assessment. Opt Express.

[118] Czupryna J, Kachur A, Blankemeyer E, Popov A, Arroyo A, Karp J, Delikatny E (2015). Cerenkov-specific contrast agents for detection of pH in vivo. J Nucl Med.

[119] D’Souza J, Hensley H, Doss M, Beigarten C, Torgov M, Olafsen T, Jian Q, Robinson M (2017). Cerenkov luminescence imaging as a modality to evaluate antibody-based PET radiotracers. J Nucl Med.

[120] Glaser A, Zhang R, Davis S, Gladstone D, Pogue B (2012). Time-gated Cherenkov emission spectroscopy from linear accelerator irradiation of tissue phantoms. Opt Lett.

[121] Glaser A, Voigt W, Davis S, Zhang R, Gladstone D, Pogue B (2013). Three-dimensional čerenkov tomography of energy deposition from ionizing radiation beams. Opt Lett.

[122] Glaser A, Zhang R, Gladstone D, Pogue B (2014). Optical dosimetry of radiotherapy beams using Cherenkov radiation: the relationship between light emission and dose. Phys Med Biol.

[123] Jang K, Yagi T, Pyeon C, Yoo W, Shin S, Jeong C, Min B, Shin D, Misawa T, Lee B (2013). Application of Cerenkov radiation generated in plastic optical fibers for therapeutic photon beam dosimetry. J Biomed Opt.

[124] Cho J, Taschereau R, Olma S, Liu K, Chen Y, Shen C, Van Dam R, Chatziioannou A (2009). Cerenkov radiation imaging as a method for quantitative measurements of beta particles in a microfluidic chip. Phys Med Biol.

[125] Ciarrocchi E, Belcari N, Cataldi G, Erba P, Del Guerra A. Quantitative Cerenkov luminescence imaging: measurements and simulations. In: 2016 IEEE Nuclear Science Symposium and Medical Imaging Conference (2016 NSS/MIC). IEEE (in press): 2016.

[126] Pagliazzi M, Ciarrocchi E, Del Guerra A, Belcari N, Boschi F, Spinelli A. Development of a simulation environment for Cerenkov luminescence imaging. In: 2013 IEEE Nuclear Science Symposium and Medical Imaging Conference (2013 NSS/MIC). IEEE: 2013. p. 1–6.

[127] Boschi F, Pagliazzi M, Spinelli A (2016). Cerenkov luminescence imaging of human breast cancer: a Monte Carlo simulations study. J Instrumentation.

[128] Altabella L, Gigliotti C, Spinelli A (2016). In vivo small animals beta detection: A Monte Carlo feasibility study from beta to Cerenkov luminescence imaging. Phys Med Eur J Med Phys.

[129] Cao X, Zhan Y, Cao X, Liang J, Chen X (2017). Harnessing the power of Cerenkov luminescence imaging for gastroenterology: Cerenkov luminescence endoscopy. Current Med Imaging Rev.

[130] Spinelli A, Schiariti M, Grana C, Ferrari M, Cremonesi M, Boschi F (2016). Cerenkov and radioluminescence imaging of brain tumor specimens during neurosurgery. J Biomed Opt.

[131] King M, Carpenter C, Sun C, Ma X, Le Q, Sunwoo J, Cheng Z, Pratx G, Xing L (2015). *β*-radioluminescence imaging: a comparative evaluation with Cerenkov luminescence imaging. J Nucl Med.

[132] James M, Gambhir S (2012). A molecular imaging primer: modalities, imaging agents, and applications. Physiol Rev.

[133] Carpenter C, Ma X, Liu H, Sun C, Pratx G, Wang J, Gambhir S, Xing L, Cheng Z (2014). Cerenkov luminescence endoscopy: Improved molecular sensitivity with *β*–emitting radiotracers. J Nucl Med.

[134] Thorek D, Das S, Grimm J (2014). Molecular imaging using nanoparticle quenchers of Cerenkov luminescence. Small.

[135] Guo W, Sun X, Jacobson O, Yan X, Min K, Srivatsan A, Niu G, Kiesewetter D, Chang J, Chen X (2015). Intrinsically radioactive [64Cu] CuIns/Zns quantum dots for PET and optical imaging: improved radiochemical stability and controllable Cerenkov luminescence. ACS Nano.

[136] Natarajan A, Habte F, Liu H, Sathirachinda A, Hu X, Cheng Z, Nagamine C, Gambhir S (2013). Evaluation of 89zr-rituximab tracer by Cerenkov luminescence imaging and correlation with PET in a humanized transgenic mouse model to image NHL. Mol Imaging Biol.

[137] Spinelli A, Boschi F, D’Ambrosio D, Calderan L, Marengo M, Fenzi A, Menegazzi M, Sbarbati A, Del Vecchio A, Calandrino R (2011). Cherenkov radiation imaging of beta emitters: in vitro and in vivo results. Nuclear Instruments Methods Phys Res Sect A Accelerators Spectrometers Detectors Assoc Equip.

[138] Lohrmann C, Zhang H, Thorek D, Desai P, Zanzonico P, O’Donoghue J, Irwin C, Reiner T, Grimm J, Weber W (2015). Cerenkov luminescence imaging for radiation dose calculation of a 90Y-labeled gastrin-releasing peptide receptor antagonist. J Nucl Med.

